# Supplementary material for: P. aeruginosa CtpA protease adopts a novel activation mechanism to initiate the proteolytic process
Source: EMBO J. 2024 Mar 11;43(8):1634–52. doi: 10.1038/s44318-024-00069-6 (PMC11021448; doi:10.1038/s44318-024-00069-6)
Supplement: Supplementary file 1 — Appendix [file 44318_2024_69_MOESM1_ESM.pdf]

APPENDIX

P. aeruginosa CtpA protease adopts a novel activation mechanism to initiate the proteolytic process

Authors: Hao-Chi Hsu, Michelle Wang, Amanda Kovach, Andrew J. Darwin, Huilin Li

Table of Contents:

Appendix Table S1	Page 2-3
Appendix Table S2	Page 4
Appendix Table S3	Page 5
Appendix Table S4	Page 6-7
References for appendix tables	Page 8
Appendix Figure S1 and legend	Page 9
Appendix Figure S2 and legend	Page 10
Appendix Figure S3 and legend	Page 11
Appendix Figure S4 and legend	Page 12
Appendix Figure S5 and legend	Page 13
Appendix Figure S6 and legend	Page 14
Appendix Figure S7 and legend	Page 15
Appendix Figure S8 and legend	Page 16
Appendix Figure S9 and legend	Page 17
Appendix Figure S10 and legend	Page 18
Appendix Figure S11 and legend	Page 19
Appendix Figure S12 and legend	Page 20
Appendix Figure S13 and legend	Page 21

Appendix Table S1. Strains and plasmids used in this study

Name	Genotype/Features	Reference or Source
***P. aeruginosa* strains**		
PAK	wild-type PAK strain	(Strom & Lory, 1986)
AJDP730	PAK Δ*ctpA*	(Srivastava *et al*, 2018)
AJDP1091	PAK Δ*lbcA::aacC1*	(Srivastava *et al.*, 2018)
***E. coli* strain**		
BL21(DE3)	F-$^-$ *ompT gal [dcm] [lon]* $hsdS_B$ (r_B^- m_B^-; *E. coli* B strain) lDE3	(Studier *et al*, 1990)
BTH101	*cya-99 araD139 galE15 galK16 rpsL* (Sm^r) *hsdR2 mcrA1 mcrB1*	Euromedex
**Plasmids**		
pET15b	Amp^r, pMB1 *ori*, *T7p* expression vector	Novagen
pCDFDuet-1	Sm/Sp^r, CDF *ori*, duel *T7p* expression vector	Novagen
pHERD26T	Tet^r, pMB1 *ori*, *araBp* expression vector	(Qiu *et al*, 2008)
pKNT25	Kan^r, p15A *ori*, vector for fuision to N-terminus of Cya-T25	(Karimova *et al*, 2005)
pUT18C	Amp^r, ColE1 *ori*, vector for fuision to C-terminus of Cya-T18	(Karimova *et al*, 1998)
pAJD3037	*araBp-ctpA* in pHERD26T	(Hsu *et al*, 2022)
pAJD3045	*araBp-ctpA-S302A* in pHERD26T	(Hsu *et al.*, 2022)
pAJD3046	*araBp-ctpA-L388A* in pHERD26T	This study
pAJD3047	*araBp-ctpA-L388M* in pHERD26T	This study
pAJD3109	*araBp-lbcA* in pHERD26T	(Hsu *et al.*, 2022)
pAJD3193	*araBp-ctpA-L46A* in pHERD26T	This study
pAJD3194	*araBp-ctpA-L46K* in pHERD26T	This study
pAJD3195	*araBp-ctpA-A50V* in pHERD26T	This study
pAJD3196	*araBp-ctpA-A50K* in pHERD26T	This study
pAJD3109	*araBp-lbcA* in pHERD26T	(Hsu *et al.*, 2022)
pAJD3197	*araBp-lbcA-L57A* in pHERD26T	This study
pAJD3198	*araBp-lbcA-L57K* in pHERD26T	This study
pAJD3199	*araBp-lbcA-V87A* in pHERD26T	This study
pAJD3200	*araBp-lbcA-V87K* in pHERD26T	This study

pAJD3217	*lbcA-L57A* in pUT18C	This study
pAJD3218	*lbcA-L57K* in pUT18C	This study
pAJD3219	*lbcA-V87A* in pUT18C	This study
pAJD3220	*lbcA-V87K* in pUT18C	This study
pAJD3221	*araBp-ctpA-H84A* in pHERD26T	This study
pAJD3222	*araBp-ctpA-G246M* in pHERD26T	This study
pAJD3223	*araBp-ctpA-F325A* in pHERD26T	This study
pAJD3224	*araBp-ctpA-K327A* in pHERD26T	This study
pAJD3225	*araBp-ctpA-Q331A* in pHERD26T	This study
pAJD3237	*ctpA-L46A-S302A* in pKNT25	This study
pAJD3238	*ctpA-L46K-S302A* in pKNT25	This study
pAJD3239	*ctpA-A50V-S302A* in pKNT25	This study
pAJD3240	*ctpA-A50K-S302A* in pKNT25	This study
pAJD3262	*araBp-ctpA-E385A* in pHERD26T	This study
pAJD3263	*araBp-ctpA-N394A* in pHERD26T	This study
pET15b_CtpAΔN37_S302A	*T7p-his$_6$-ctpAΔN37_S302A* in pET15b	(Hsu *et al.*, 2022)
pET15b_CtpAΔN37_ΔC6	*T7p-his$_6$-ctpAΔN37_ ΔC6* in pET15b	(Hsu *et al.*, 2022)
pCDF_LbcAΔN31	*T7p-lbcAΔN31* in pCDFDuet-1	This study
pET15b_CtpAΔN37_E385A	*T7p-his$_6$-ctpAΔN37_E385A* in pET15b	This study
pET15b_CtpAΔN37_L388A	*T7p-his$_6$-ctpAΔN37_ L388A* in pET15b	This study
pET15b_CtpAΔN37_ E385AL388A	*T7p-his$_6$-ctpAΔN37_ E385AL388A* in pET15b	This study
pET15b_CtpAΔN37_ΔTRL	*T7p-his$_6$-ctpAΔN37_ ΔTRL* in pET15b	This study
pET24b_LbcAΔN31	*T7p-lbcAΔN31* in pET24b	This study

Appendix Table S2. Cryo-EM data collection, refinement, and validation statistics

	CtpA-LbcA (Local structure)	Local CtpA-LbcA (Upper TPR position)	Local CtpA-LbcA (Lower TPR position)	CtpA-LbcA (C3)
**EMBD**	EMD-40849	EMD-40850	EMD-40851	EMD-40852
**PDB code**	8SXE	8SXF	8SXG	8SXH
**Data collection and processing**				
Magnification	105,000x	105,000x	105,000x	105,000x
Voltage (kV)	300	300	300	300
Electron exposure (e^{-}/Å)	66	66	66	66
Defocus range (μm)	-1.3 to -1.8	-1.3 to -1.8	-1.3 to -1.8	-1.3 to -1.8
Pixel size (Å)	0.828	0.828	0.828	0.828
Symmetry imposed	C1	C1	C1	C3
Initial particle images (no.)	2,090,314	2,090,314	2,090,314	2,090,314
Final particle images (no.)	623,456	326,300	297,156	334,527
Map resolution (Å)	3.55	3.92	4.14	3.94
FSC threshold	0.143	0.143	0.143	0.143
Map resolution range (Å)	2.7 - 5.1	2.8 – 6.8	3.0 – 7.0	2.7 – 6.5
**Refinement**				
Initial model used	7RQF, 7RQH	7RQF, 7RQH	7RQF, 7RQH	7RQF, 7RQH
Model resolution (Å)	3.8	4.0	4.2	4.1
FSC threshold	0.5	0.5	0.5	0.5
Map-sharpening B-factor ($Å^2$)	-133	-135	-165	-196
Model composition				
Non-hydrogen atoms	8,285	11,321	11,372	19,719
Protein residues	1,101	1,477	1,483	2,625
B-factors ($Å^2$)				
Protein	63	72.9	115	76.4
R.m.s. deviations				
Bond lengths (Å)	0.002	0.002	0.002	0.003
Bond angles (°)	0.465	0.479	0.498	0.455
**Validation**				
MolProbity score	1.60	1.74	1.69	1.68
Clashscore	6	9	9	7
Poor rotamers (%)	0.6	0.6	0.4	0.6
Ramachandran statistics (%)				
Favored	96.39	96.44	96.72	95.8
Allowed	3.61	3.56	3.28	4.2
Outliers	0	0	0	0

Appendix Table S3. Cryo-EM data collection, refinement, and validation statistics for EM maps without model building

	**CtpA-LbcA (C1)**	**CtpA-LbcA (Upper TPR position)**	**CtpA-LbcA (Lower TPR position)**
**EMBD code**	EMD-40848	EMD-40847	EMD-40846
**Data collection and processing**			
Magnification	105,000x	105,000x	105,000x
Voltage (kV)	300	300	300
Electron exposure (e^-/Å)	66	66	66
Defocus range (µm)	-1.3 to -1.8	-1.3 to -1.8	-1.3 to -1.8
Pixel size (Å)	0.828	0.828	0.828
Symmetry imposed	C1	C1	C1
Initial particle images (no.)	2,090,314	2,090,314	2,090,314
Final particle images (no.)	623,456	326,300	297,156
Map resolution (Å)	3.84	4.04	4.26
FSC threshold	0.143	0.143	0.143
Map resolution range (Å)	2.7 – 6.7	2.7 – 6.1	2.7 – 7.2
**Refinement**			
Map-sharpening B-factor ($Å^2$)	-162	-155	-165

Appendix Table S4. Primers used in this study

Name	
For protein production and purification plasmids	
CtpA ∆TIL, F383-R412	GAG CAG CTT CGA GGG CTT C<br>CG CAG GAT TCC GAC TAC CA
CtpA 385A	CTG CAG GTC GGC TGC CTT GAA GCC CTC<br>GAG GGC TTC AAG GCA GCC GAC CTG CAG
CtpA 388A	CAG GTG GCC CTG CGC GTC GGC TTC CTT G<br>CAA GGA AGC CGA CGC GCA GGG CCA CCT G
CtpA 385A/388A	GGT GGC CCT GCG CGT CGG CTG CCT TGA AGC CC<br>GGG CTT CAA GGC AGC CGA CGC GCA GGG CCA CC
LbcA E32-K182	
Vector	GAG CTC GGC GCG CCT GCA
Vector	GAA TTC GGC CGC CGC GTT AG
	CTA ACG CGG CGG CCG AAT TCG AAG ACA CCG CGG TCG AG
	CCT GCA GGC GCG CCG AGC TCC TAT TTC TTC AAC AGG TGG TCG
For *in vivo* CtpA activity assay pHERD26T plasmid derivatives	
CtpA F EcoRI	CCGGAATTCACAGGGATAGGCGCCGAACTCG
CtpA R XbaI	GGCTCTAGAGCAACGCGGAACGCCGACATCAG
CtpA L46A F	GCCCCTCGACGAGGCGCGTACCTTCGCC
CtpA L46A R	GGCGAAGGTACGCGCCTCGTCGAGGGGC
CtpA L46K F	GCCCCTCGACGAGAAGCGTACCTTCGCC
CtpA L46K R	GGCGAAGGTACGCTTCTCGTCGAGGGGC
CtpA A50V F	CTGCGTACCTTCGTCGAGGTTCTCGAC
CtpA A50V R	GTCGAGAACCTCGACGAAGGTACGCAG
CtpA A50K F	GCTGCGTACCTTCAAGGAGGTTCTCGACC
CtpA A50K R	GGTCGAGAACCTCCTTGAAGGTACGCAGC
CtpA H84A F	GCAACCTCGACCCGGCCTCCGCCTACCTCG
CtpA H84A R	CGAGGTAGGCGGAGGCCGGGTCGAGGTTGC
CtpA G246M F	GGACTGCAGCACGCCCATGGGGTTGTTGCGCAG
CtpA G246M R	CTGCGCAACAACCCCATGGGCGTGCTGCAGTCC
CtpA F325A F	TGGGCACCGACAGCGCCGGCAAGGGCTCGG
CtpA F325A R	CCGAGCCCTTGCCGGCGCTGTCGGTGCCCA

CtpA K327A F	CCGACAGCTTCGGCGCGGGCTCGGTGCAGA
CtpA K327A R	TCTGCACCGAGCCCGCGCCGAAGCTGTCGG
CtpA Q331A F	GCAAGGGCTCGGTGGCGACCGTGCTGCCGC
CtpA Q331A R	GCGGCAGCACGGTCGCCACCGAGCCCTTGC
CtpA L388A F	CAAGGAAGCCGACGCGCAGGGCCACCTG
CtpA L388A R	CAGGTGGCCCTGCGCGTCGGCTTCCTTG
CtpA L388M F	CAAGGAAGCCGACATGCAGGGCCACCTG
CtpA L388M R	CAGGTGGCCCTGCATGTCGGCTTCCTTG
LbcA F EcoRI	CCGGAATTCGCCACCCTCGCAGGTTCAAG
LbcA R HindIII	GGCCAAGCTTCAAGGAGTCTCGGCGCCGGTCAGG
LbcA L57A F	CCTGTATTCGCTGGCGGTGGCCGAACTG
LbcA L57A R	CAGTTCGGCCACCGCCAGCGAATACAGG
LbcA L57K F	CCTGTATTCGCTGAAGGTGGCCGAACTGG
LbcA L57K R	CAGTTCGGCCACCTTCAGCGAATACAGG
LbcA V87A F	CGCGACCCCGGGGCGTCCGAGCGCGCCT
LbcA V87A R	AGGCGCGCTCGGACGCCCCGGGGTCGCG
LbcA V87K F	CCGCGACCCCGGGAAGTCCGAGCGCGCCT
LbcA V87K R	AGGCGCGCTCGGACTTCCCGGGGTCGCGG

To amplify *ctpA* and *lbcA* variants for cloning into pUT18C and pKNT25 two hybrid plasmids

ctpA TH F XbaI	GGCTCTAGATGCCGACGCCCCGGCCGACG
ctpA TH R KpnI	CGGGGTACCTTGCCGCGGGTGACGCTCAG
lbcA TH F XbaI	GGCTCTAGACCAGTCACTTATCCACAAGAC
lbcA TH R KpnI	CGGGGTACCGGAGTCTCGGCGCCGGTCAG

References for appendix tables

Hsu HC, Wang M, Kovach A, Darwin AJ, Li H (2022) *Pseudomonas aeruginosa* C-Terminal Processing Protease CtpA Assembles into a Hexameric Structure That Requires Activation by a Spiral-Shaped Lipoprotein-Binding Partner. *mBio* 13: e0368021

Karimova G, Dautin N, Ladant D (2005) Interaction network among *Escherichia coli* membrane proteins involved in cell division as revealed by bacterial two-hybrid analysis. *J Bacteriol* 187: 2233-2243

Karimova G, Pidoux J, Ullmann A, Ladant D (1998) A bacterial two-hybrid system based on a reconstituted signal transduction pathway. *Proc Natl Acad Sci USA* 95: 5752-5756

Qiu D, Damron FH, Mima T, Schweizer HP, Yu HD (2008) PBAD-based shuttle vectors for functional analysis of toxic and highly regulated genes in *Pseudomonas* and *Burkholderia* spp. and other bacteria. *Applied and environmental microbiology* 74: 7422-7426

Srivastava D, Seo J, Rimal B, Kim SJ, Zhen S, Darwin AJ (2018) A Proteolytic Complex Targets Multiple Cell Wall Hydrolases in *Pseudomonas aeruginosa*. *mBio* 9: pii: e00972-00918

Strom MS, Lory S (1986) Cloning and expression of the pilin gene of *Pseudomonas aeruginosa* PAK in *Escherichia coli*. *J Bacteriol* 165: 367-372

Studier FW, Rosenberg AH, Dunn JJ, Dubendorff JW (1990) Use of T7 RNA polymerase to direct expression of cloned genes. *Methods in Enzymology* 185: 60-89

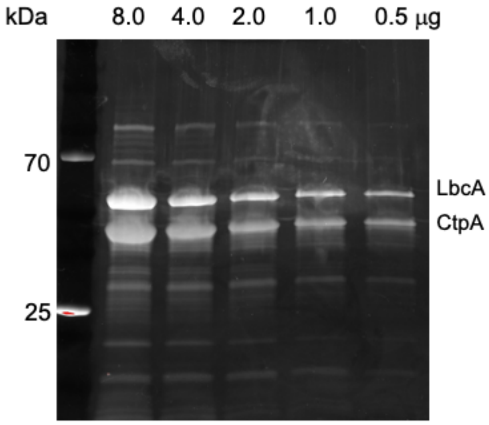

Appendix Figure S1. SYPRO Ruby stained SDS-PAGE gel of the purified CtpA–LbcA complex.
The estimated molar ratio between CtpA and LbcA is 2:1.

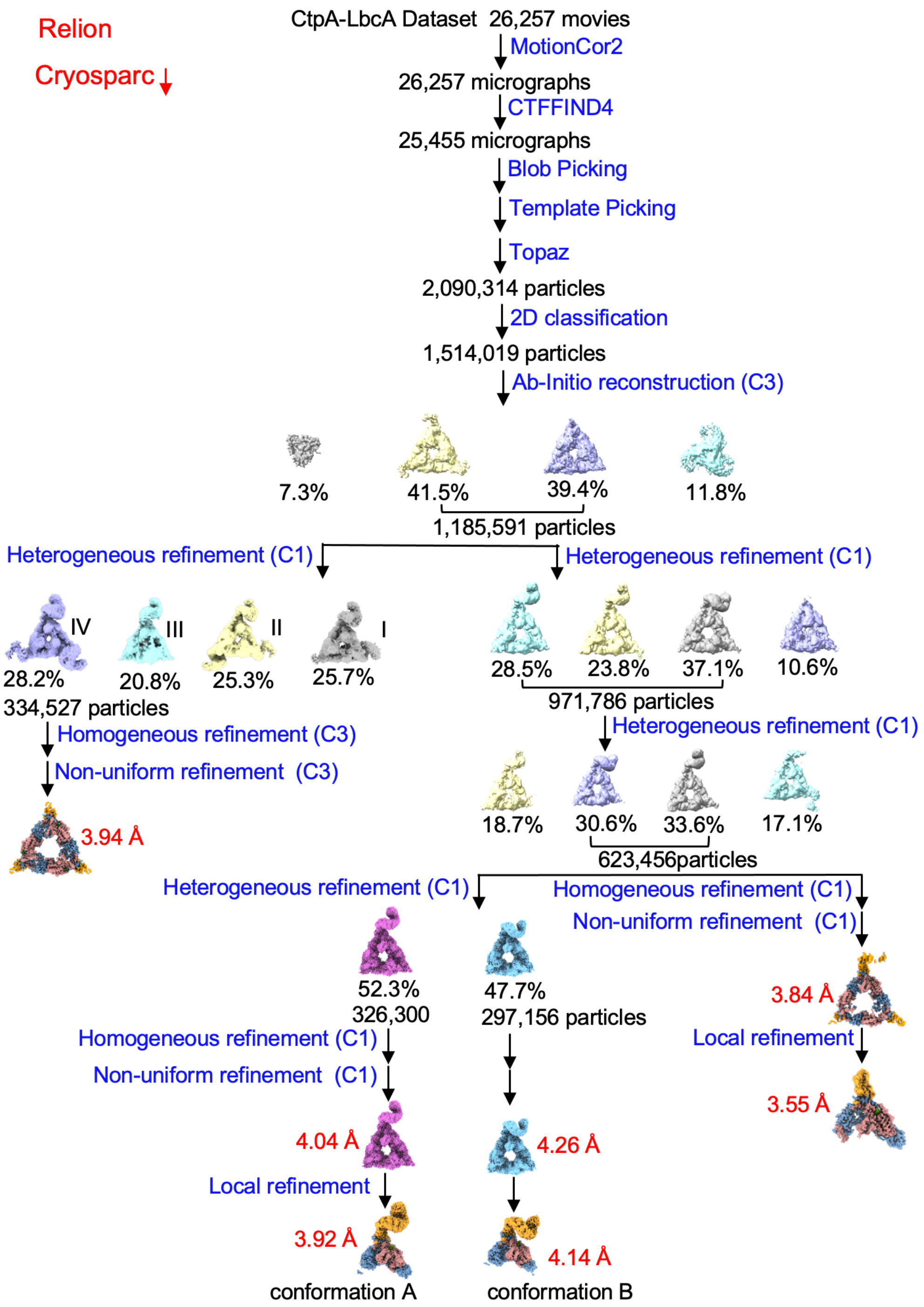

Appendix Figure S2. The flowchart of cryo-EM data processing. A combination of heterogeneous, homogenous, and local refinement resulted in a 3.84-Å resolution overall map of the LbcA-CtpA complex, a 3.55-Å resolution EM map of the LbcA adaptor domain bound a CtpA dimer in which in CtpA is activated and the other remain inactive, a three-fold symmetrical LbcA-CtpA complex at 3.94 Å resolution in which all three LbcA are bound to the same side of the CtpA hexamer, and two conformations of LbcA-bound CtpA dimer in which the LbcA is in an remote position (3.92 Å resolution) and a proximal position (4.14 Å resolution). The five EM maps presented in the manuscript are colored by individual subunits.

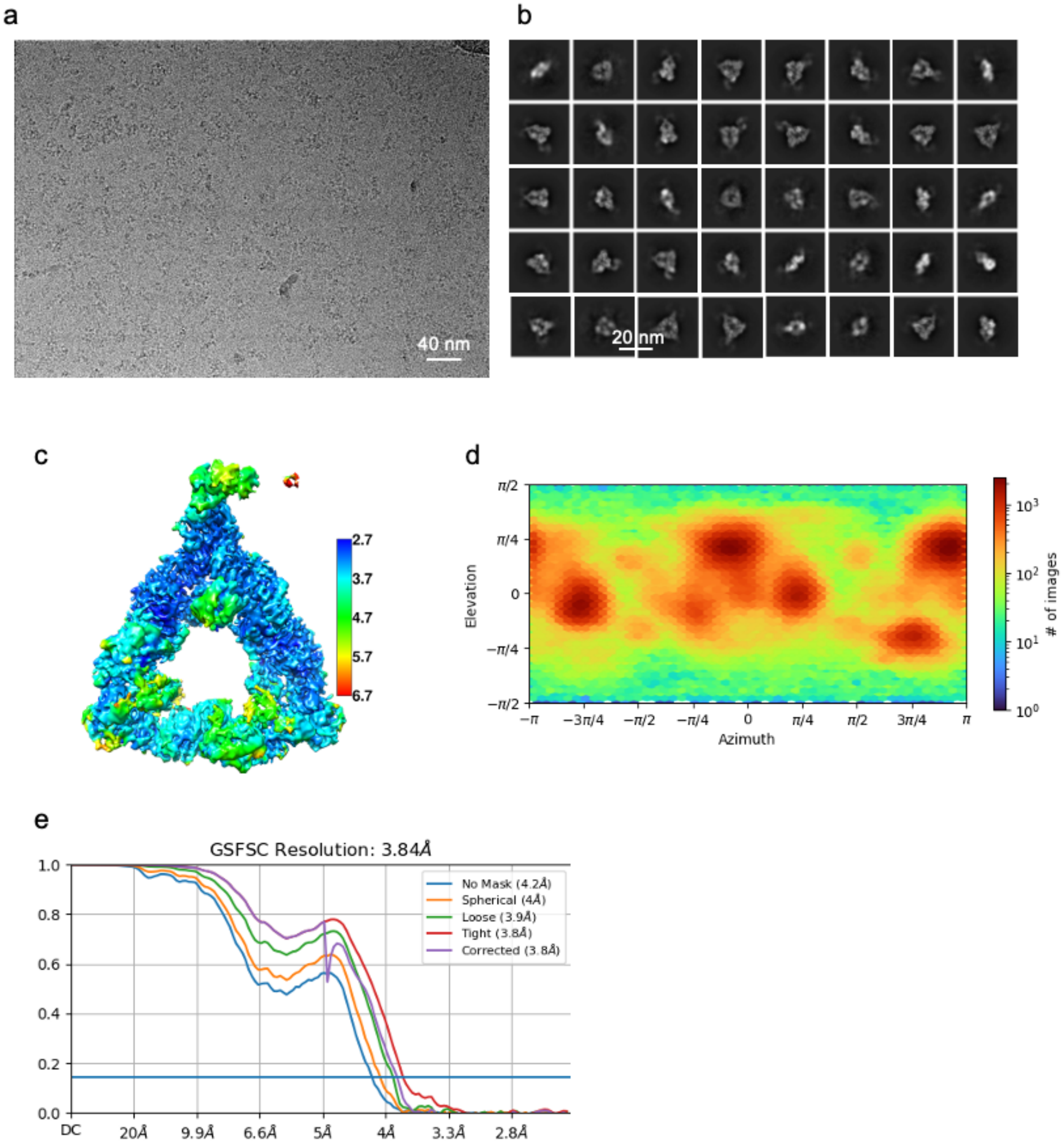

Appendix Figure S3. Cryo-EM analysis of the CtpA–LbcA complex. a) A typical raw micrograph after motion correction. A total of 26,257 raw micrographs were recorded. **b)** Selected 2D class averages showing the particles are present in various views. **c)** The overall map of CtpA–LbcA complex surface rendered and colored by the local resolution estimation. **d)** The angular distribution of particles used in the final reconstruction. **e)** Gold standard Fourier shell correction of the EM map with or without various masks.

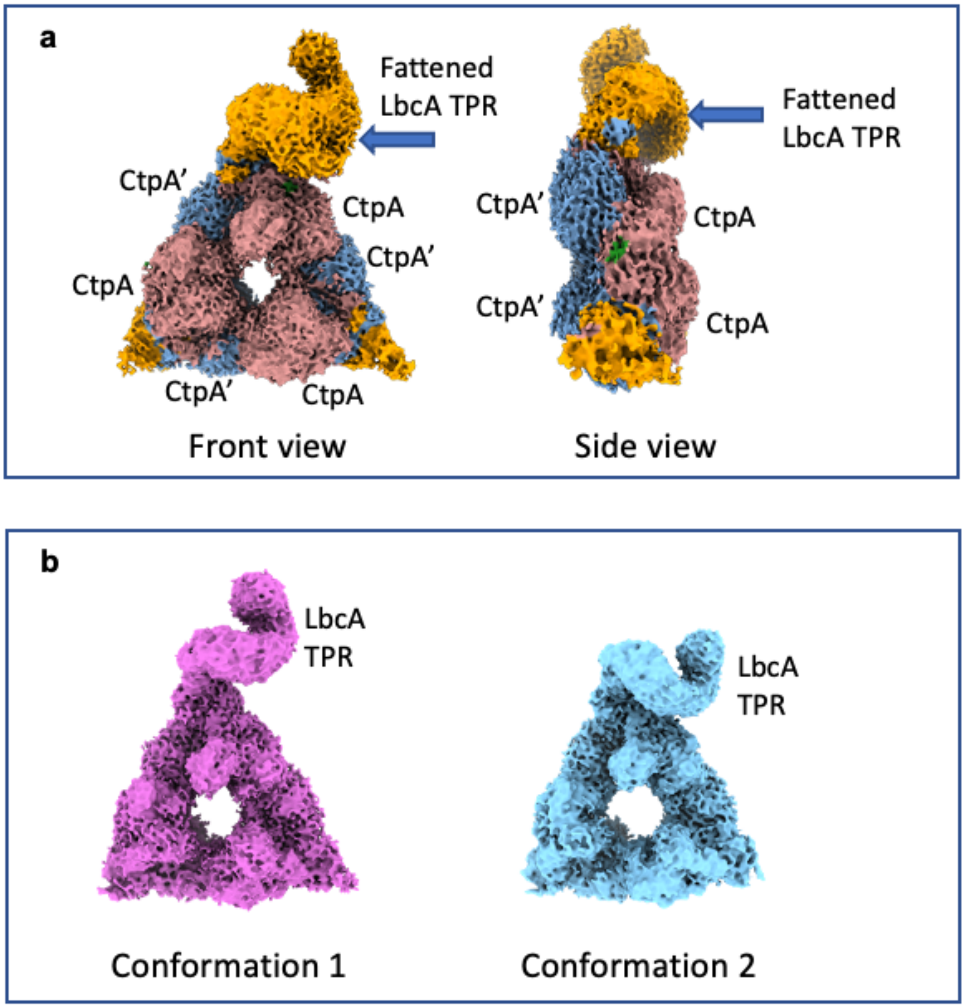

Appendix Figure S4. Demonstrations that LbcA can bind CtpA in multiple configurations. a) The 3.84 Å resolution overall EM map of CtpA–LbcA complex displayed at a low threshold. At the low threshold, the LbcA TPR domain is visible but is fattened in the middle region, indicative of co-existence of multiple configurations. **b)** Further classification focusing on the LbcA region led to two alternative conformations in which the LbcA TPR can be at a remote (left, magenta, 3.92 Å resolution) or a proximal (right, cyan, 4.14 Å resolution) positions.

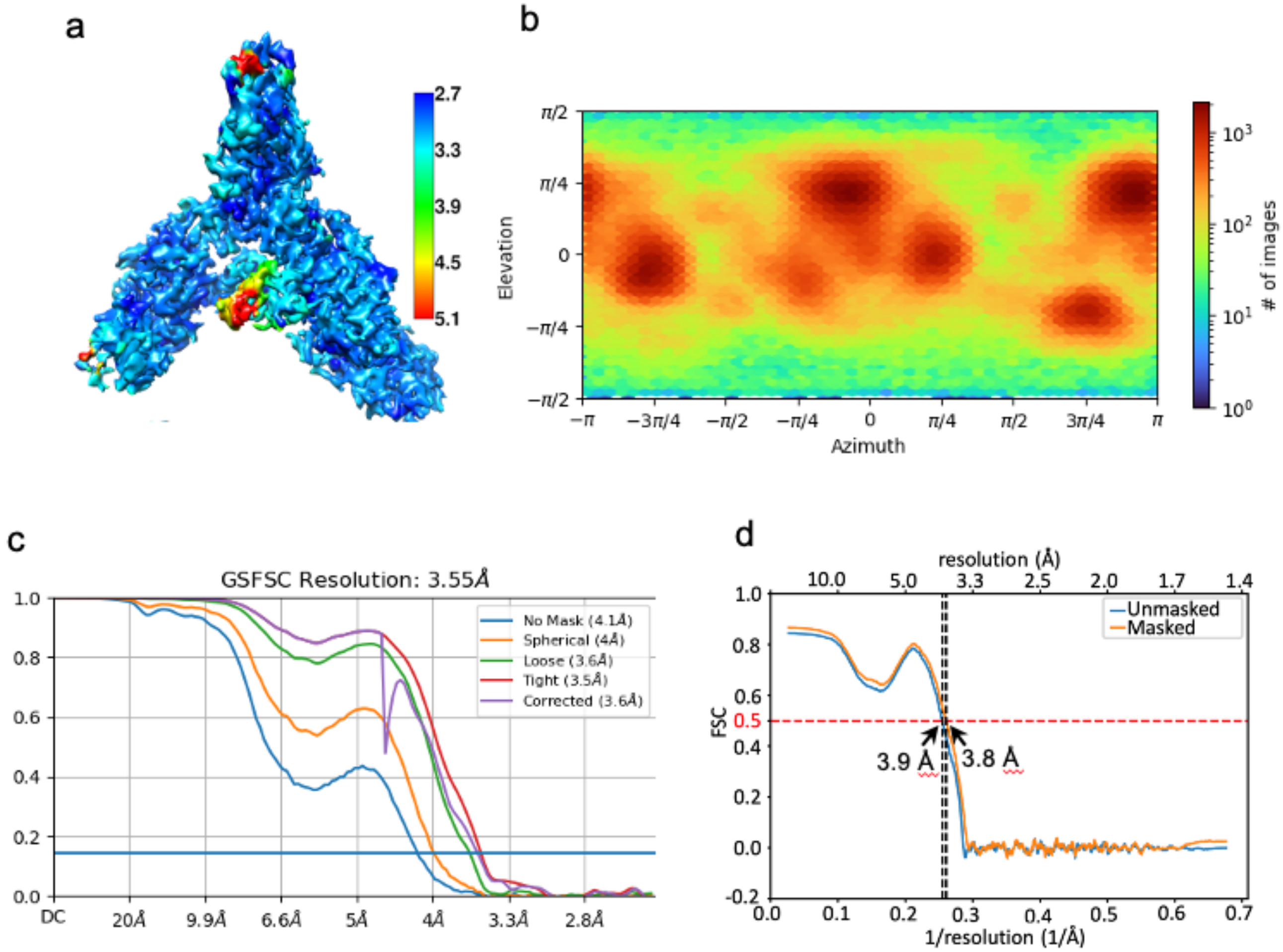

Appendix Figure S5. Locally refined CtpA–LbcA subcomplex. a) The locally refined EM map of the CtpA–LbcA complex surface rendered and colored by the local resolution estimation. **b)** The angular distribution of particles used in the final reconstruction. **c)** Gold standard Fourier shell correction of the local EM map with or without various masks. **d)** The calculated model-map FSC curves by Mtriage in the Phenix.

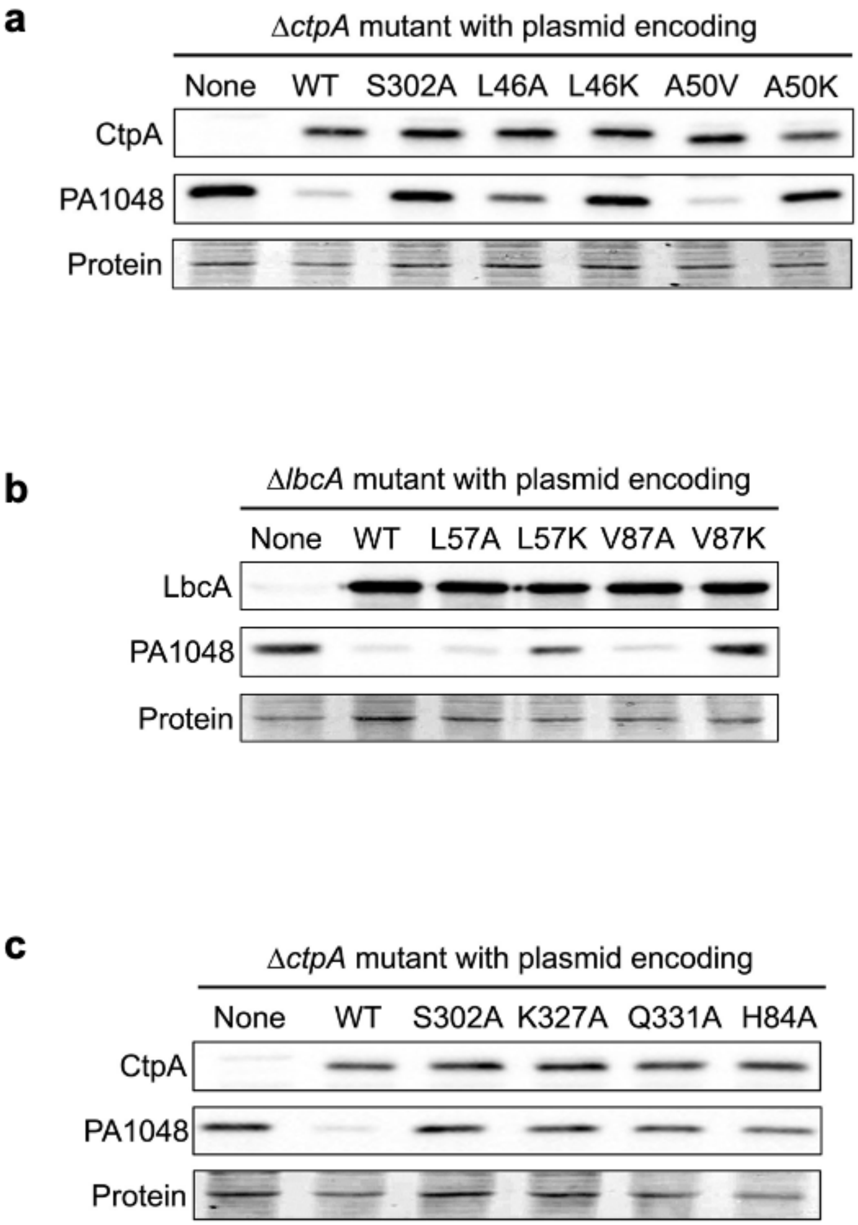

Appendix Figure S6. Effects of CtpA and LbcA mutations on PA1048 substrate levels. a) CtpA mutations that affect interaction with LbcA. b) LbcA mutations that affect interaction with CtpA. c) CtpA mutations that reduce catalytic activity. CtpA, LbcA and PA1048 were detected by immunoblot with polyclonal antisera; loading was monitored by Ponceau S total protein staining of the nitrocellulose membrane used for detection (protein). This is a single experiment to demonstrate that the mutant defects are not specific to the model substrate used in all experiments, PA1198.

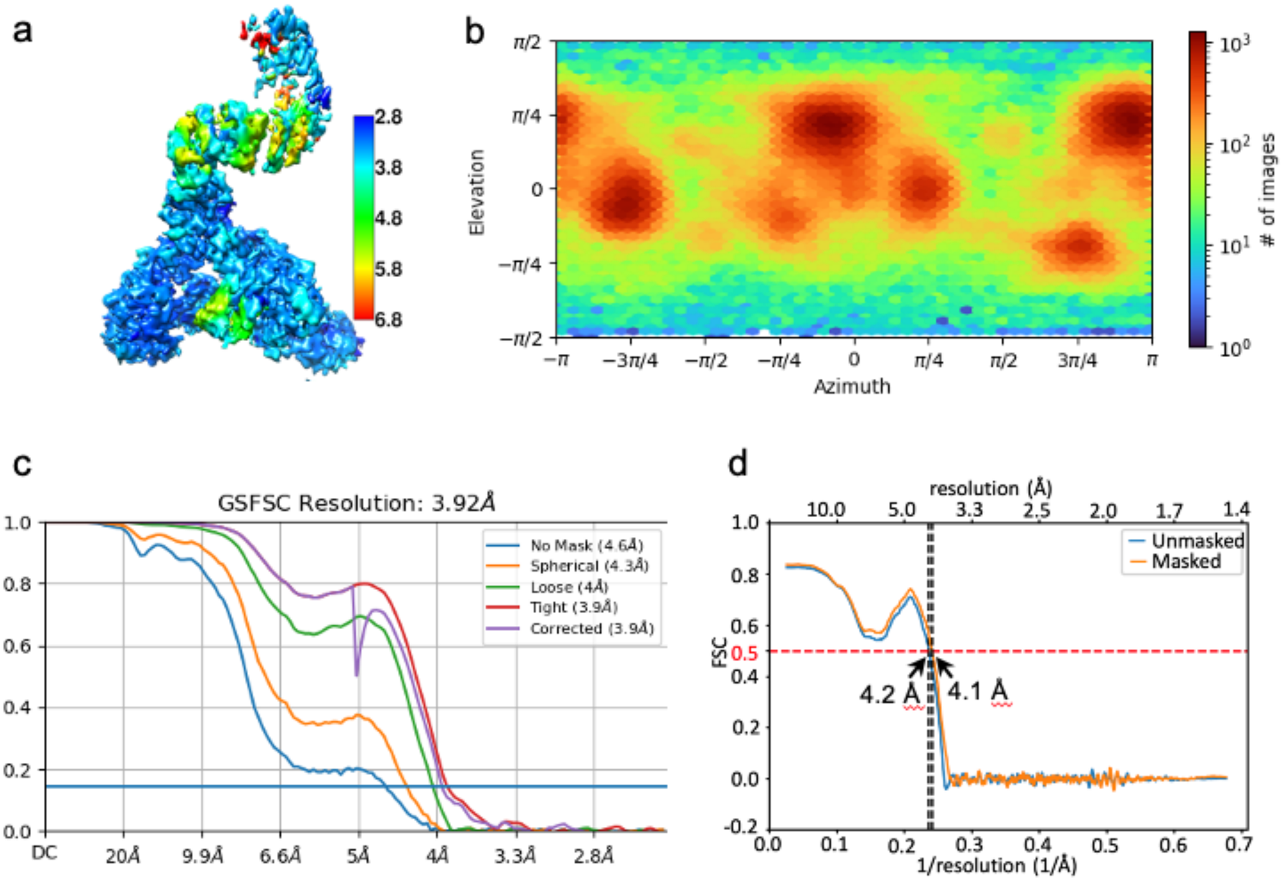

Appendix Figure S7. Locally refined EM map of the CtpA–LbcA complex with the LbcA TPR domain in the upper (remote) position. a) The local resolution estimation of locally refined EM map of the CtpA–LbcA complex with TPR domain in the remote position. **b**) The angular distribution of particles used in the final reconstruction. **c**) Gold standard Fourier shell correction of the local EM map with or without various masks. **d**) The calculated model-map FSC curves by Mtriage in the Phenix.

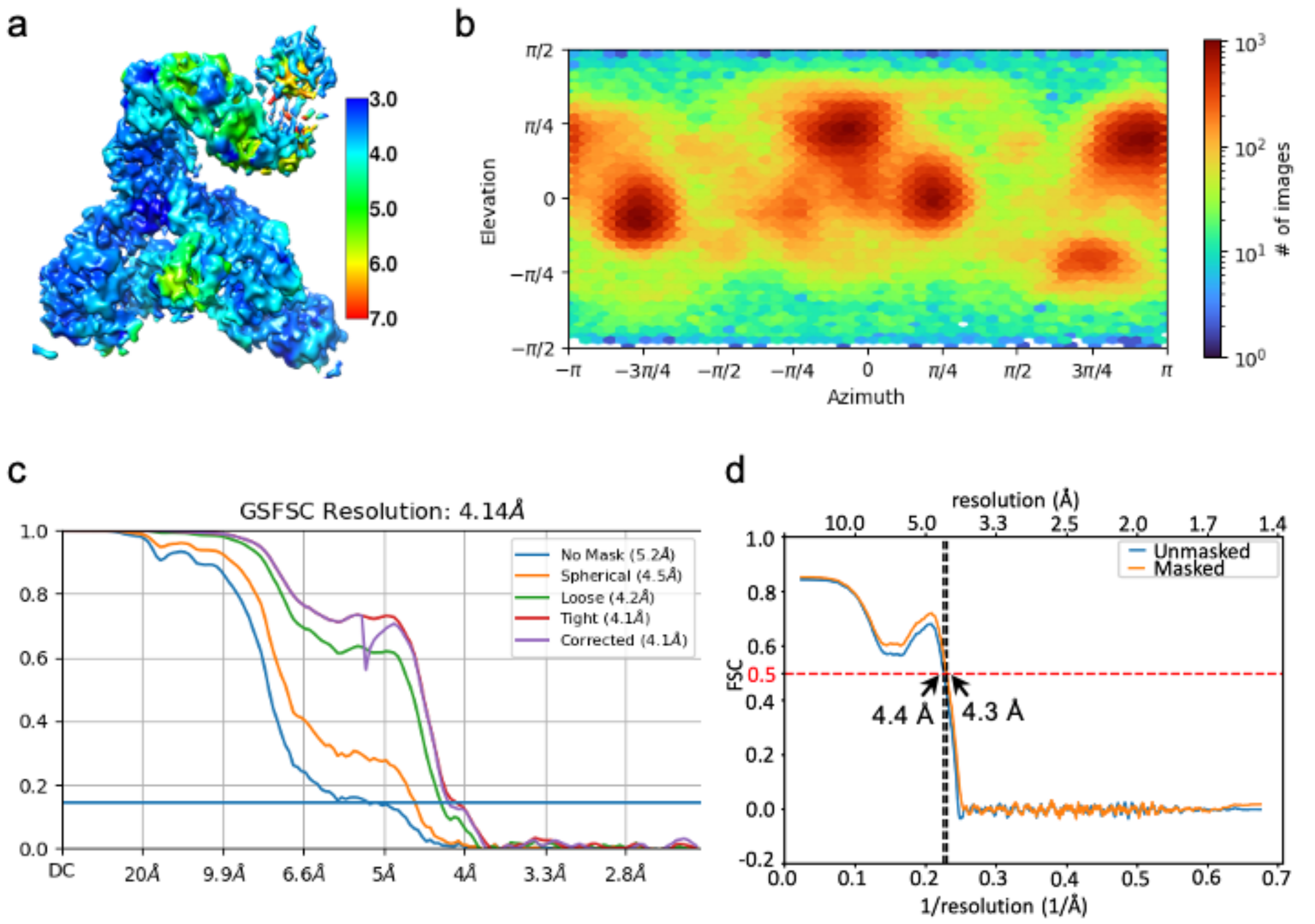

Appendix Figure S8. Locally refined EM map of the CtpA–LbcA complex with LbcA TPR domain in the lower (proximal) position. a) The local resolution estimation of locally refined EM map of the CtpA–LbcA complex with TPR domain in the proximal position. **b**) The angular distribution of particles used in the final reconstruction. **c**) Gold standard Fourier shell correction of the local EM map with or without various masks. **d**) The calculated model-map FSC curves by Mtriage in the Phenix.

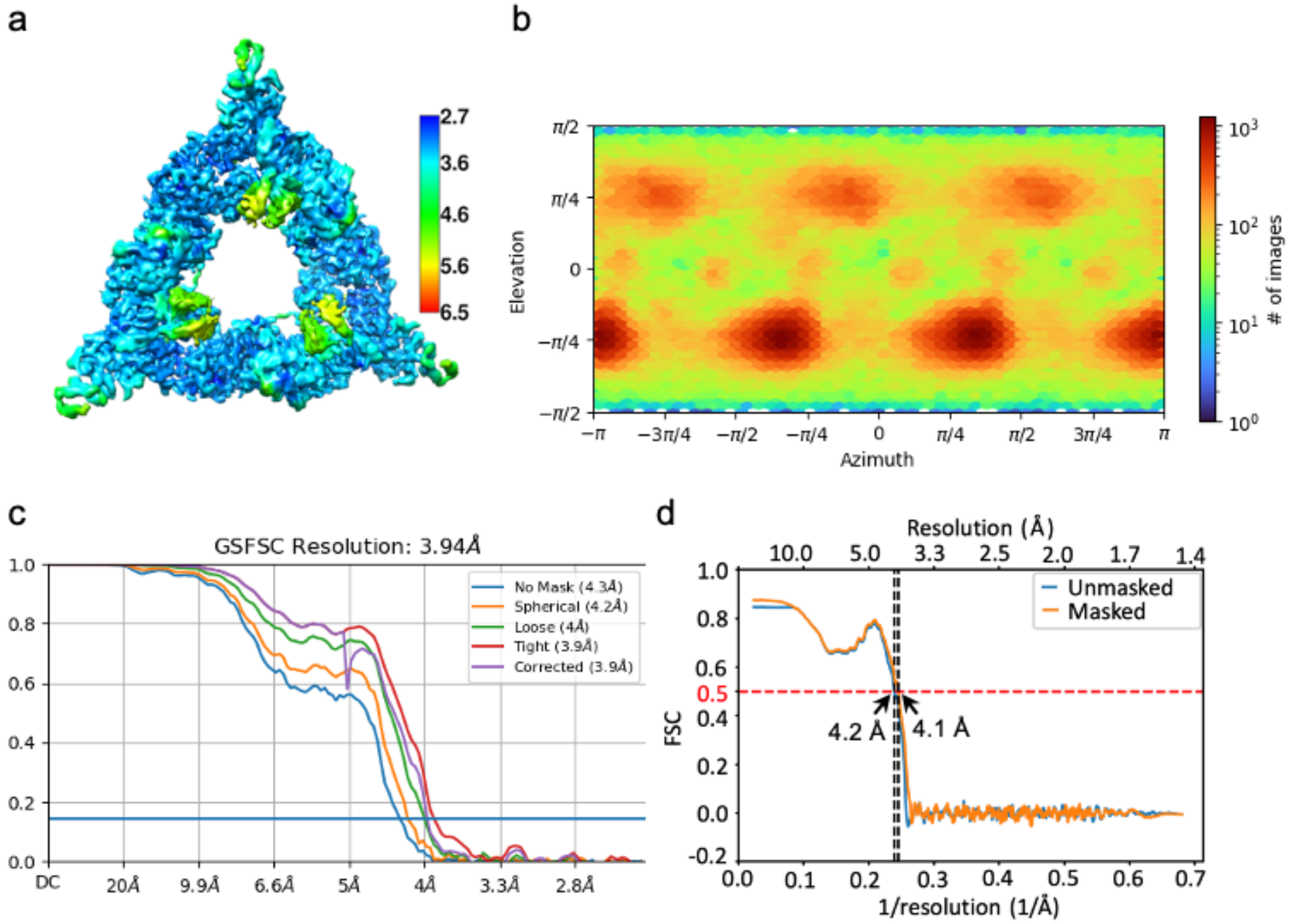

Appendix Figure S9. Three-fold symmetric EM map of the CtpA-LbcA complex. a) The local resolution estimation of C3 map derived from particles in 3D Class IV. **b**) The angular distribution of particles used in the final reconstruction. **c**) Gold standard Fourier shell correction of the local EM map with or without various masks. **d**) The calculated model-map FSC curves by Mtriage in the Phenix.

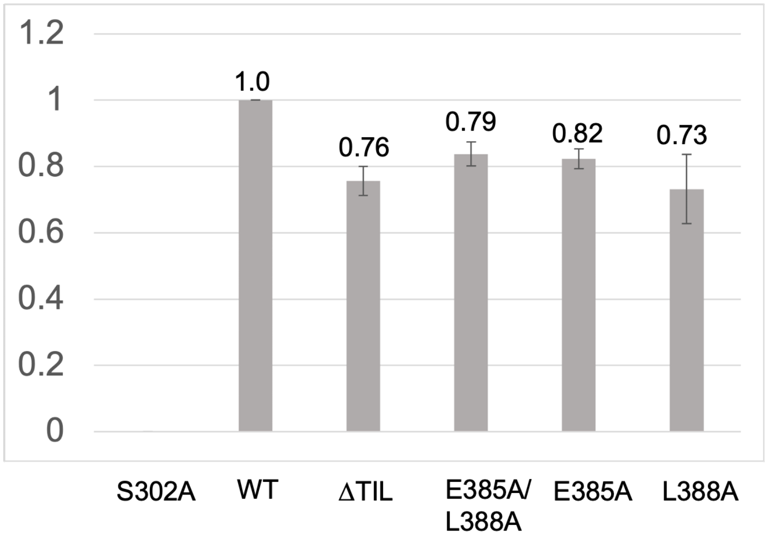

Appendix Figure S10. The relative activities of CtpA TIL mutants. The amount of the substrate protein PA1198 treated with various TIL mutant CtpA enzymes was normalized against the protein amount treated with the wild type CtpA and was used as the relative activity. The activity of S302A mutant was set to zero and the activity of wild type CtpA was set to 1. The amount of PA1198 in Fig. 6g was quantified with ImageJ. The average and standard deviation of each mutant protein were calculated from three independent experiments.

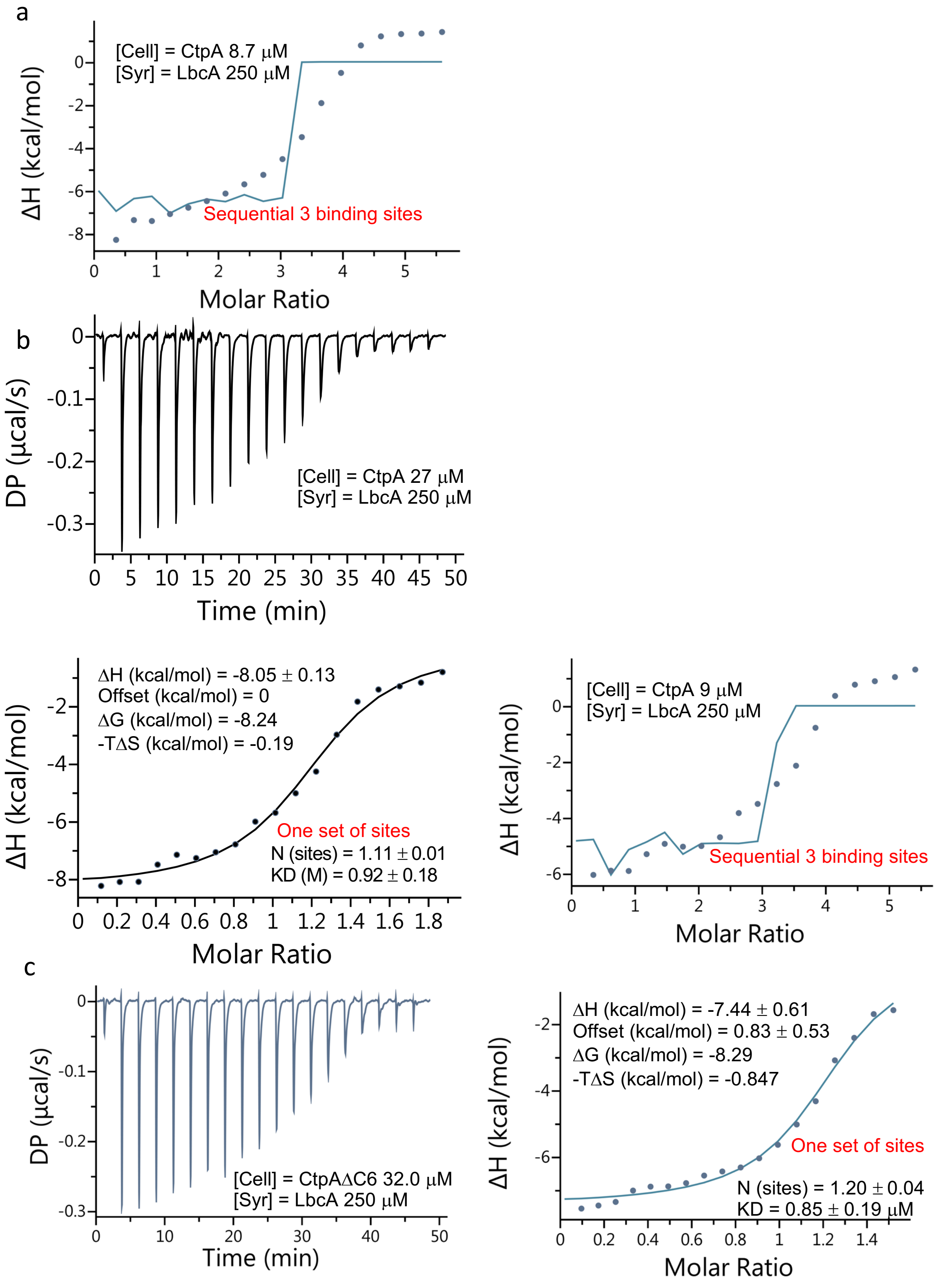

Appendix Figure S11. ITC measurement of LbcA binding to CtpA. a) Titrating LbcA into CtpAS302A. The curve does not fit with a sequential 3-site binding model. **b**) Titrating LbcA into WT CtpA. The curve was fitted with one-site (lower left) and sequential 3-site binding (lower right) model, respectively. **c**) Titrating LbcA into CtpAΔC6. The curve was fitted with the one-site binding model.

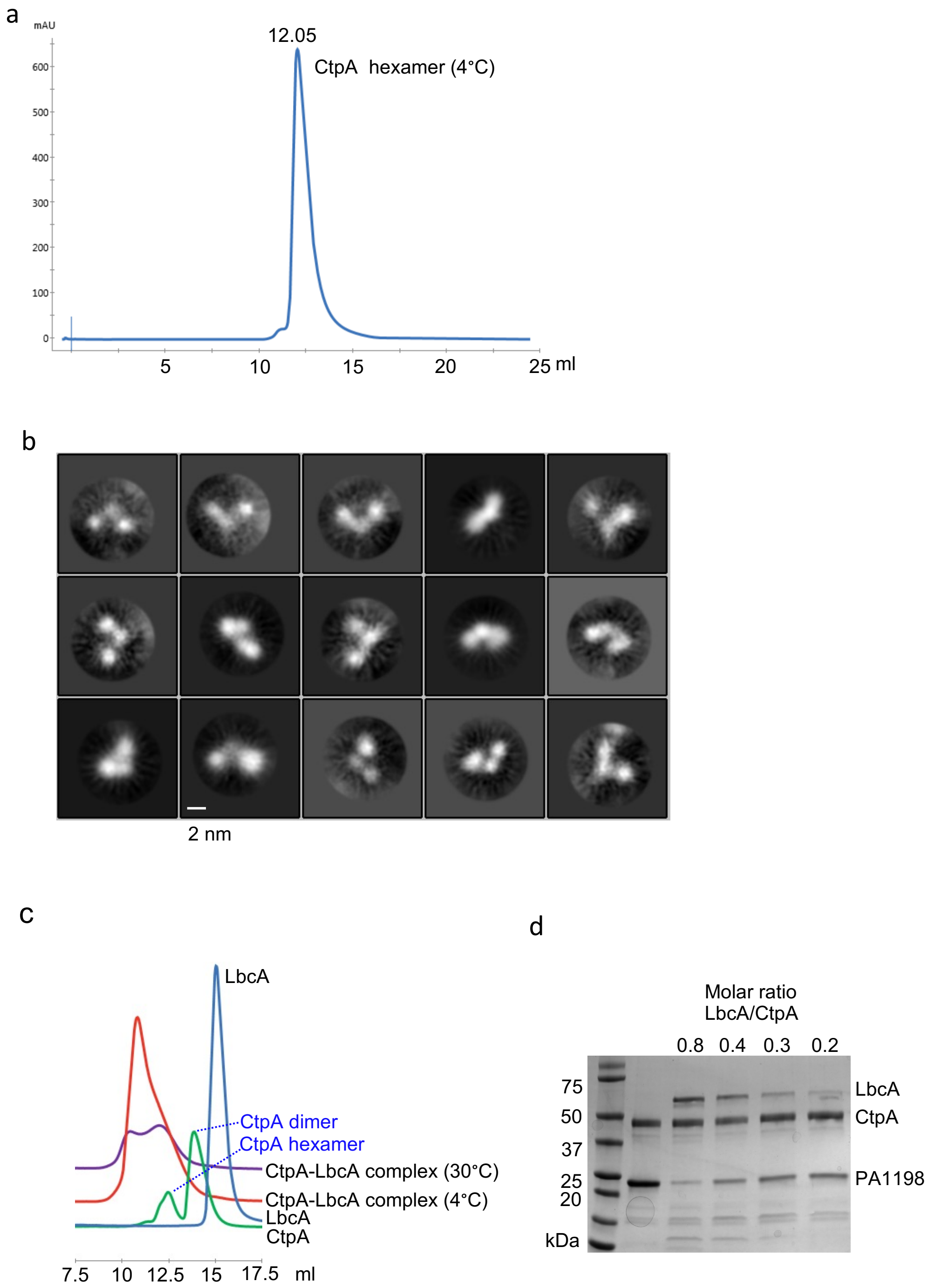

Appendix Figure S12. CtpA and LbcA assemble a temperature-sensitive complex of a CtpA hexamer bound by three LbcA. a) Gel filtration profile of CtpA at 4°C is consistent with a hexameric CtpA. **b)** 2D class averages of negatively stained CtpA revealing CtpA dimers. **c) Comparison of** the gel filtration profiles of purified CtpA (green), LbcA (light blue), and purified co-expressed CtpA–LbcA complex at 4°C (red) and 30°C (purple). CtpA and LbcA assemble primarily as the $CtpA_6LbcA_3$ complex (one CtpA hexamer bound by three LbcA) at 4°C, but its profile broadens at 30°C. **d)** Effect of LbcA on the in vitro CtpA activity. CtpA activity increases with an increasing amount of LbcA.

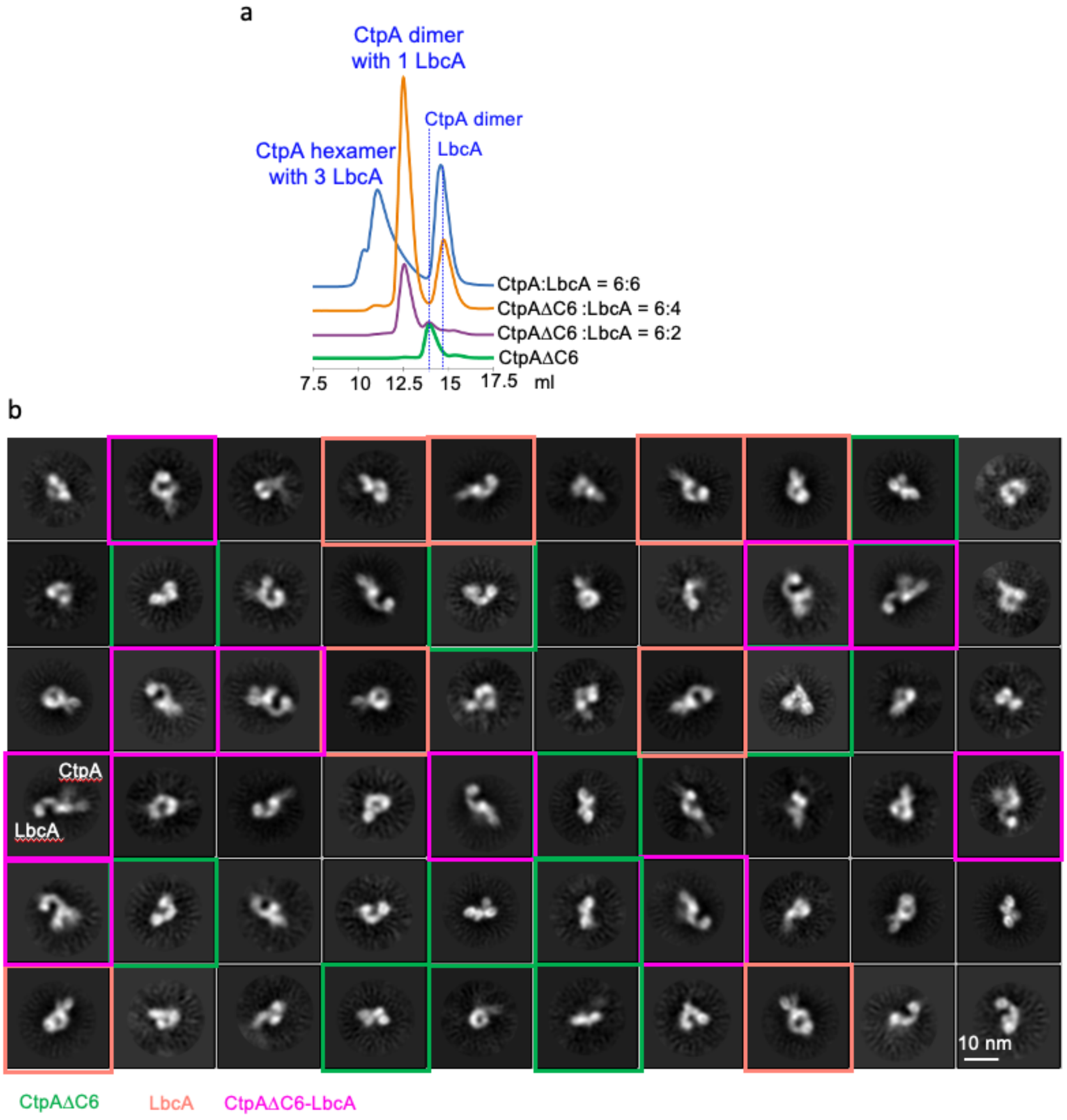

Appendix Figure S13. The C-terminal truncated CtpA (CtpAΔC6) is a dimer and binds to only one LbcA. a) The gel filtration profiles of dimeric CtpAΔC6 mixed with LbcA after incubation at 30°C. **b**) 2D class averages of cryo-EM images of the CtpAΔC6–LbcA complex, revealing mostly the dimeric CtpAΔC6 bound to one LbcA. Example averages of the dimeric CtpA ΔC6, monomeric LbcA, and the complex of a dimeric CtpAΔC6 bound to one LbcA are marked by the green, orange, and magenta squares, respectively.
