# Supplementary material for: P. aeruginosa CtpA protease adopts a novel activation mechanism to initiate the proteolytic process
Source: EMBO J. 2024 Mar 11;43(8):1634–52. doi: 10.1038/s44318-024-00069-6 (PMC11021448; doi:10.1038/s44318-024-00069-6)
Supplement: Supplementary file 2 — Source Data Fig. 1 [file 44318_2024_69_MOESM2_ESM.zip › Figure-1/Fig1b/reademe.rtf]

The cropped region is in rectangle.
