# Supplementary figures and images for: P. aeruginosa CtpA protease adopts a novel activation mechanism to initiate the proteolytic process
Source: EMBO J. 2024 Mar 11;43(8):1634–52. doi: 10.1038/s44318-024-00069-6 (PMC11021448; doi:10.1038/s44318-024-00069-6)

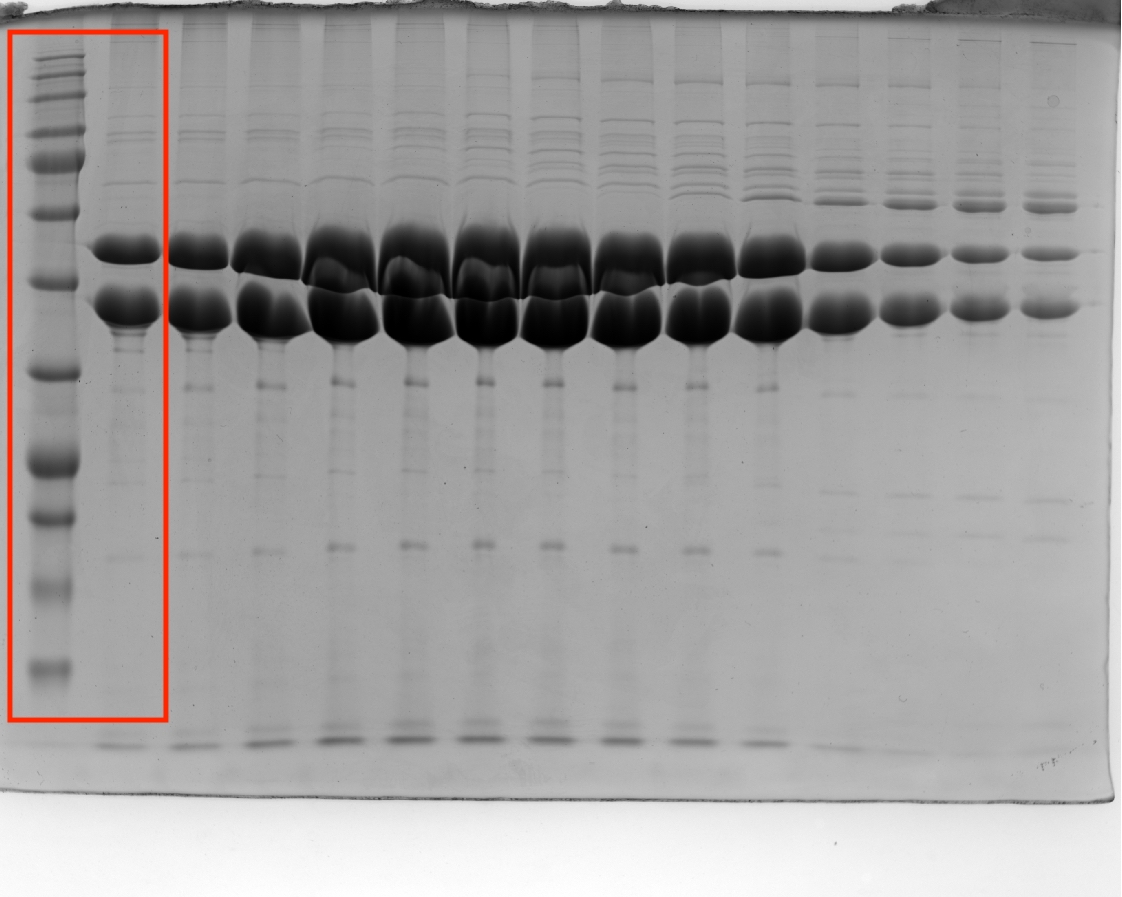

Supplement: Supplementary file 2 — Source Data Fig. 1 [file 44318_2024_69_MOESM2_ESM.zip › Figure-1/Fig1b/Fig1b.jpg]

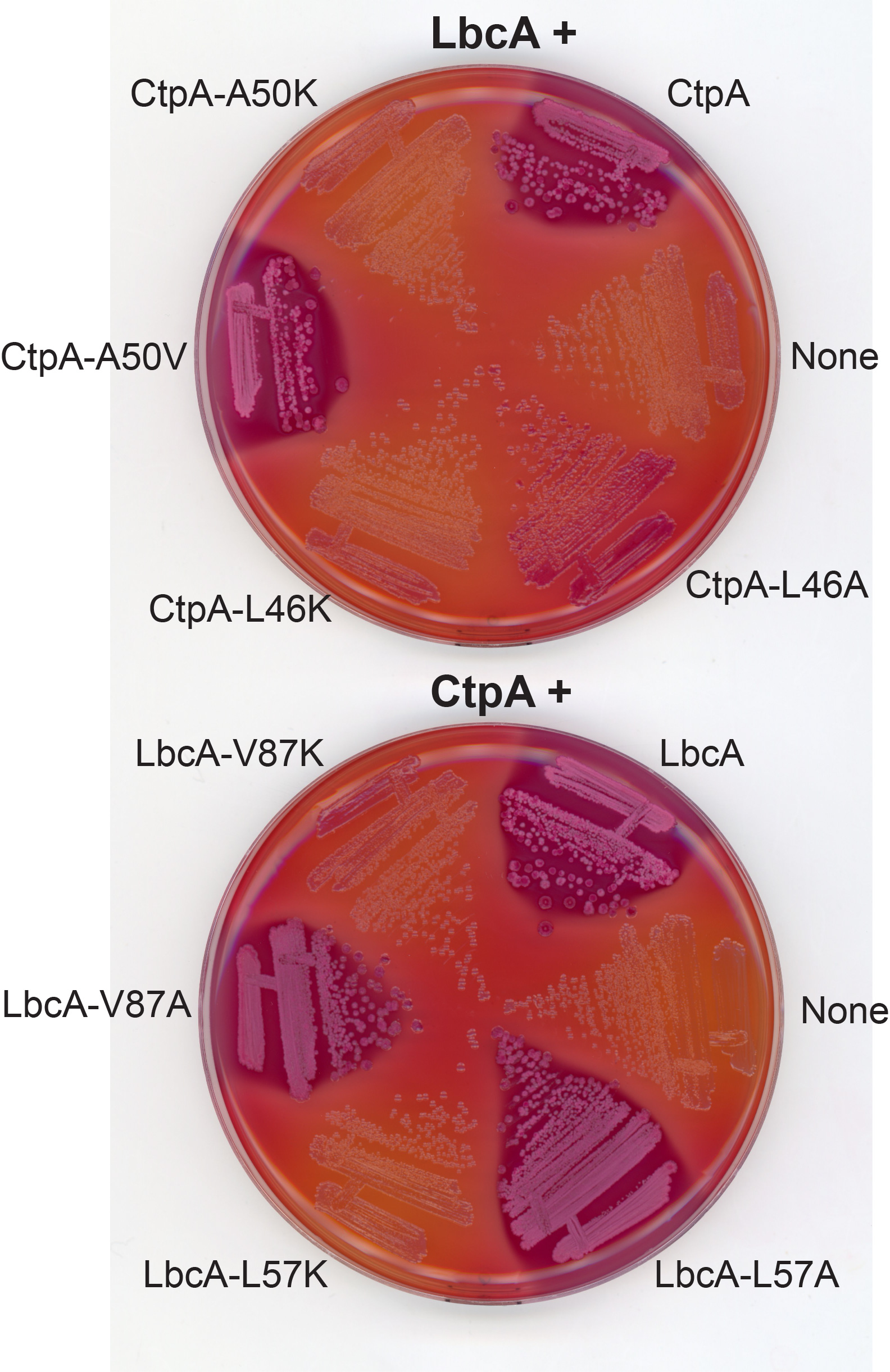

Supplement: Supplementary file 3 — Source Data Fig. 2 [file 44318_2024_69_MOESM3_ESM.zip › Figure-2/2j/Bacterial two hybrid plates.jpg]

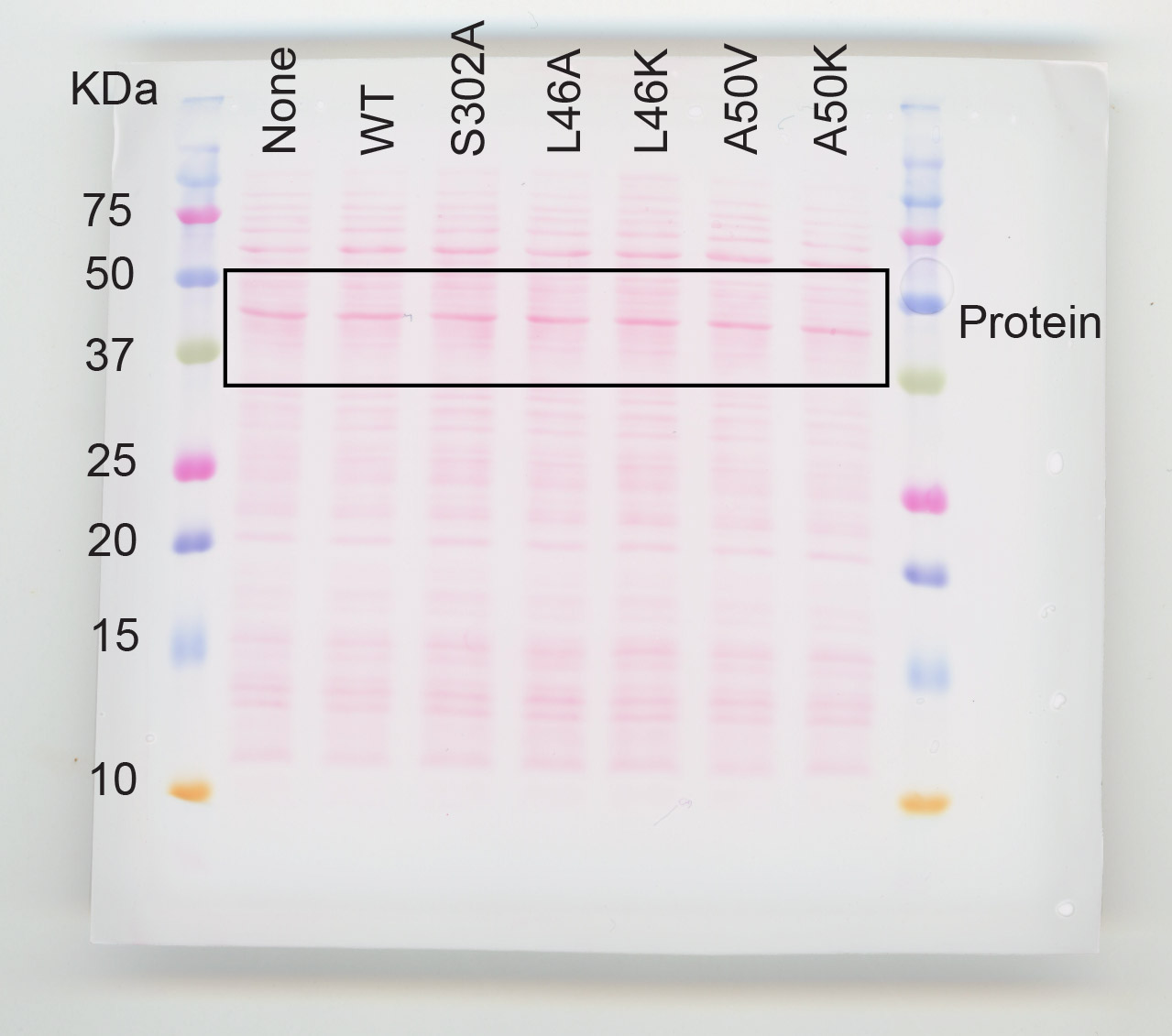

Supplement: Supplementary file 3 — Source Data Fig. 2 [file 44318_2024_69_MOESM3_ESM.zip › Figure-2/2i/CtpA mutants/Protein.jpg]

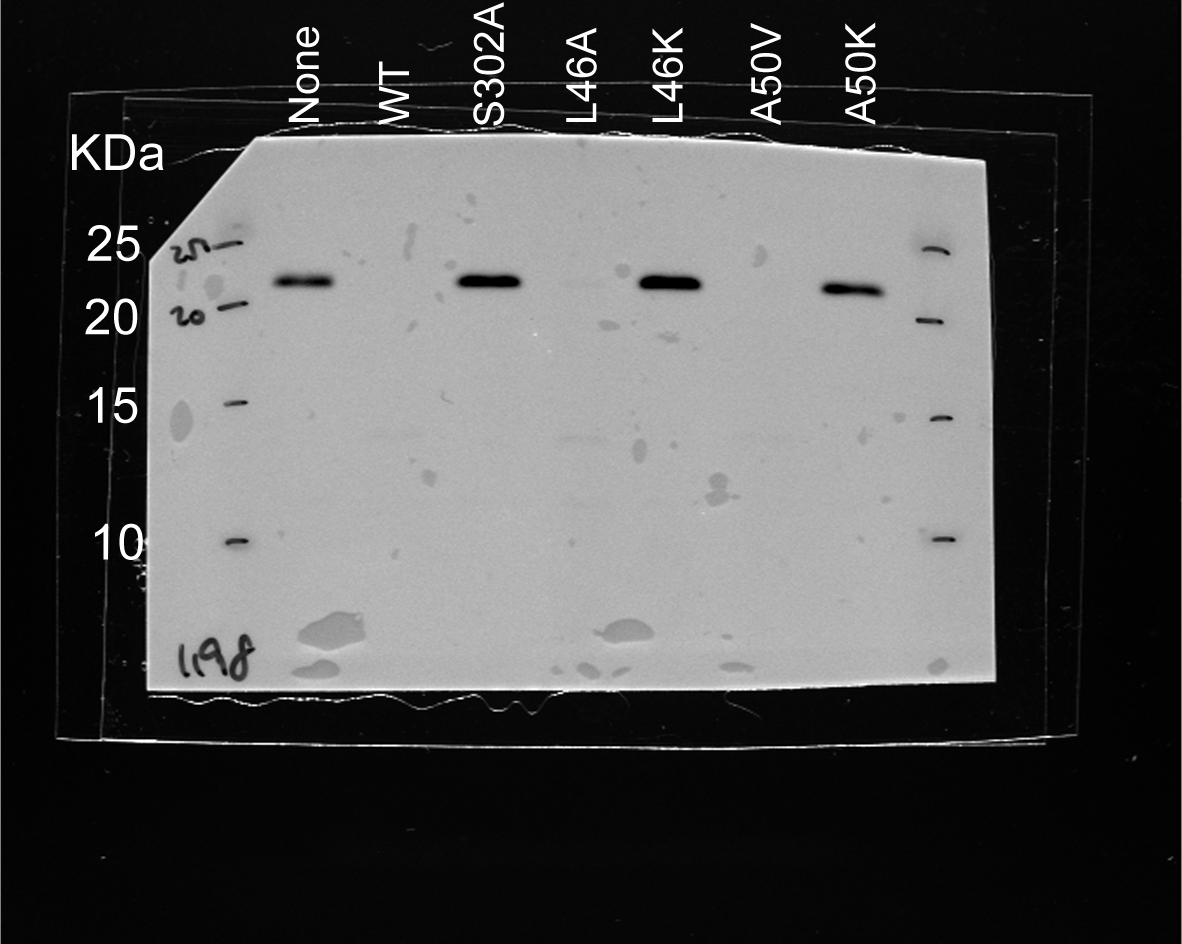

Supplement: Supplementary file 3 — Source Data Fig. 2 [file 44318_2024_69_MOESM3_ESM.zip › Figure-2/2i/CtpA mutants/PA1198 western ECL+visual overlay.tif]

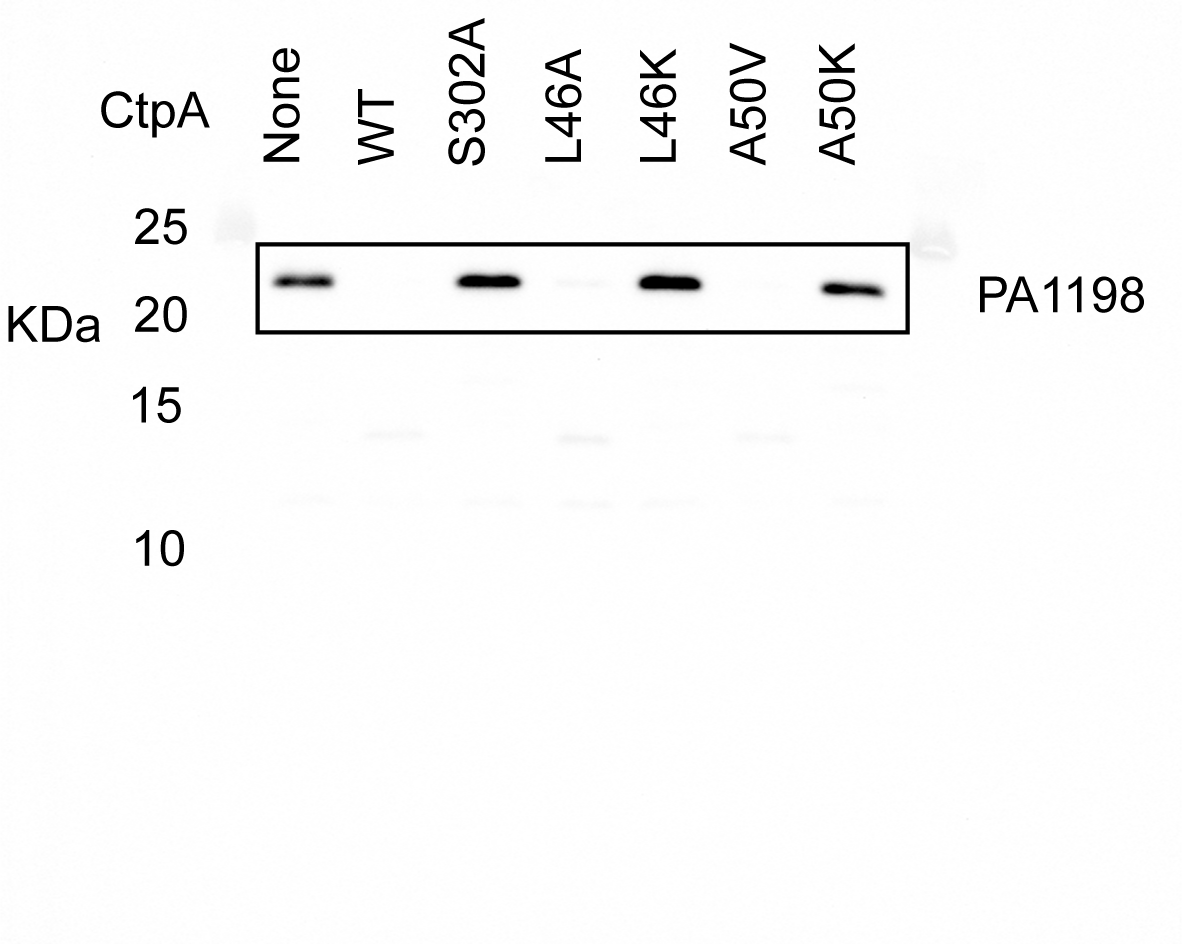

Supplement: Supplementary file 3 — Source Data Fig. 2 [file 44318_2024_69_MOESM3_ESM.zip › Figure-2/2i/CtpA mutants/PA1198 western ECL only (used for figure).tif]

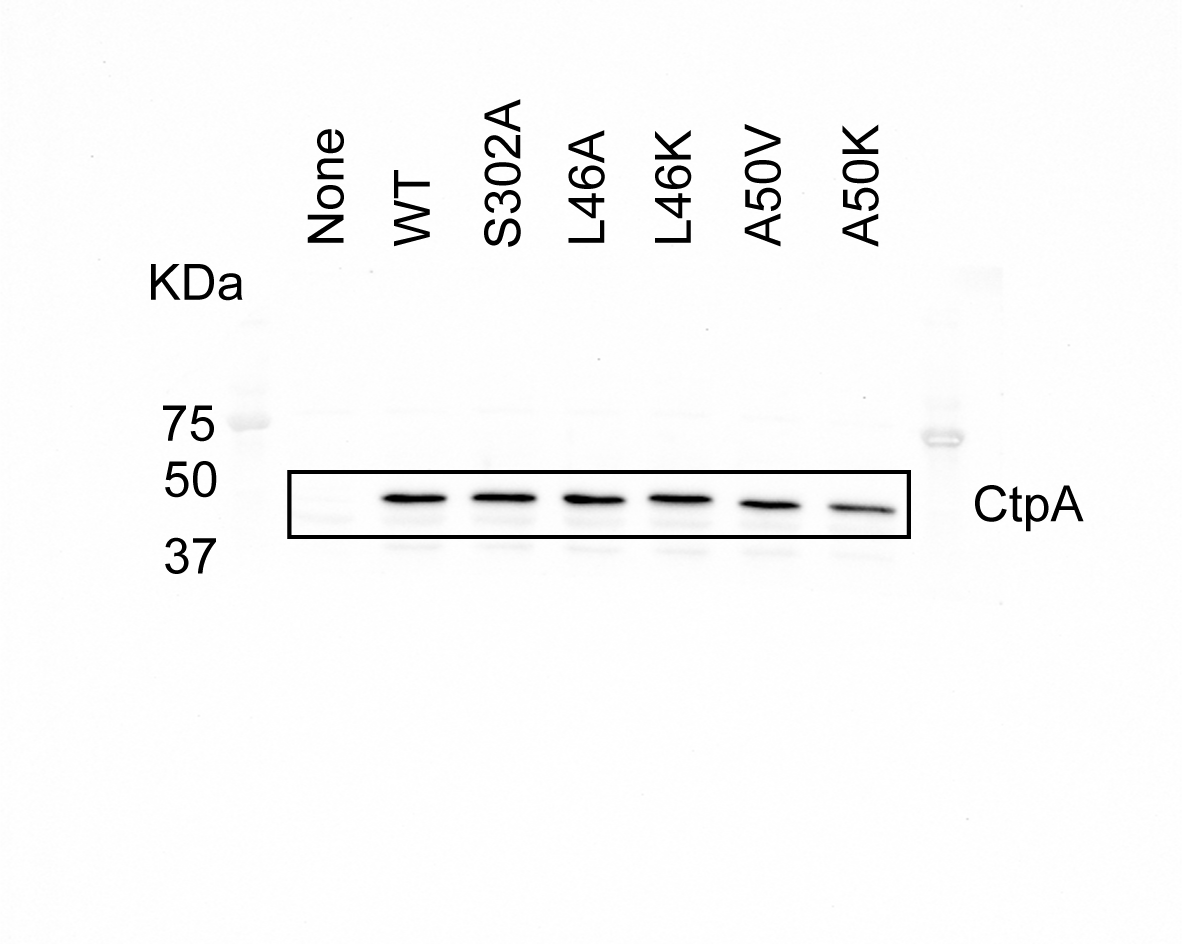

Supplement: Supplementary file 3 — Source Data Fig. 2 [file 44318_2024_69_MOESM3_ESM.zip › Figure-2/2i/CtpA mutants/CtpA western ECL only (used for figure).tif]

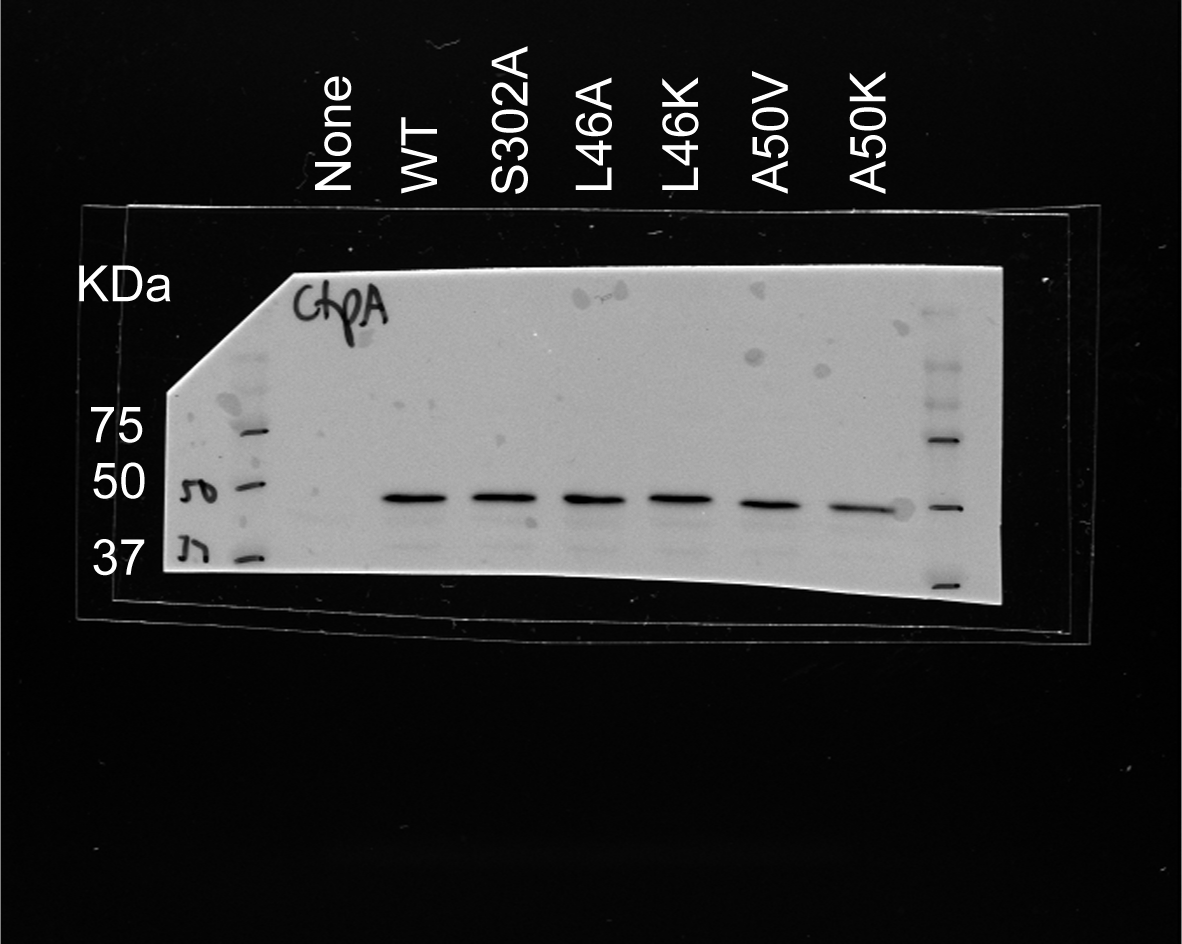

Supplement: Supplementary file 3 — Source Data Fig. 2 [file 44318_2024_69_MOESM3_ESM.zip › Figure-2/2i/CtpA mutants/CtpA western ECL+visual overlay.tif]

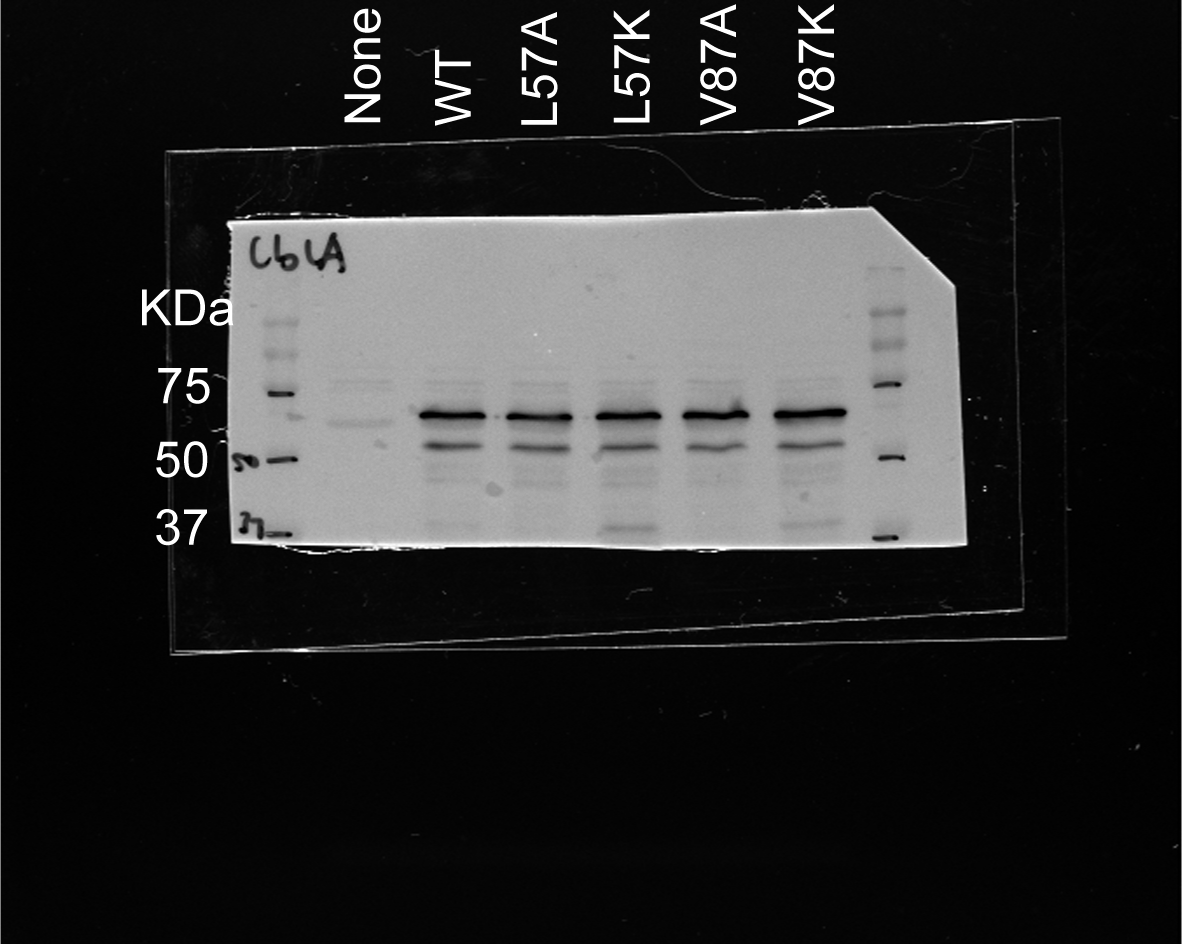

Supplement: Supplementary file 3 — Source Data Fig. 2 [file 44318_2024_69_MOESM3_ESM.zip › Figure-2/2i/LbcA mutants/LbcA western ECL+visual overlay.tif]

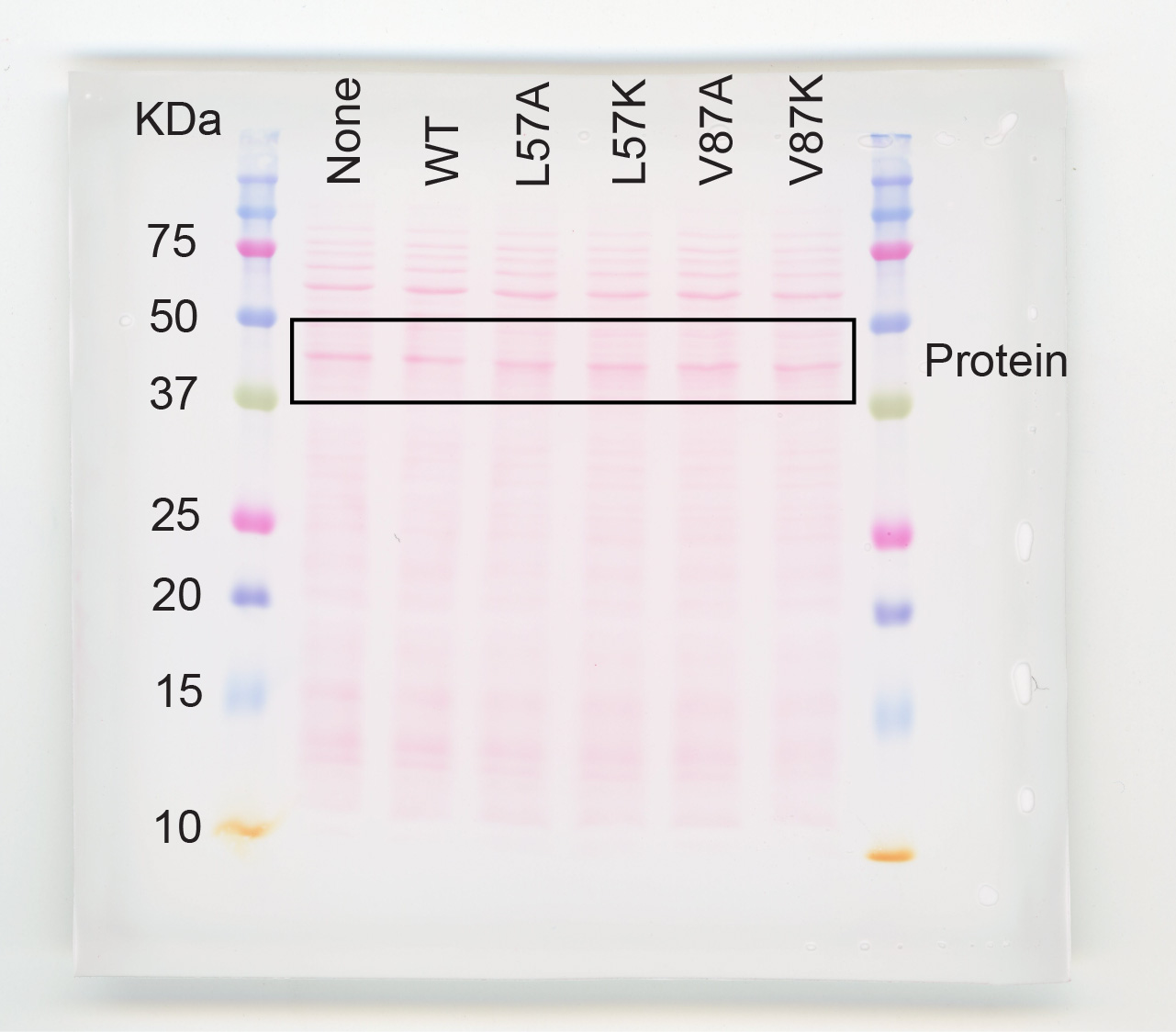

Supplement: Supplementary file 3 — Source Data Fig. 2 [file 44318_2024_69_MOESM3_ESM.zip › Figure-2/2i/LbcA mutants/Protein.jpg]

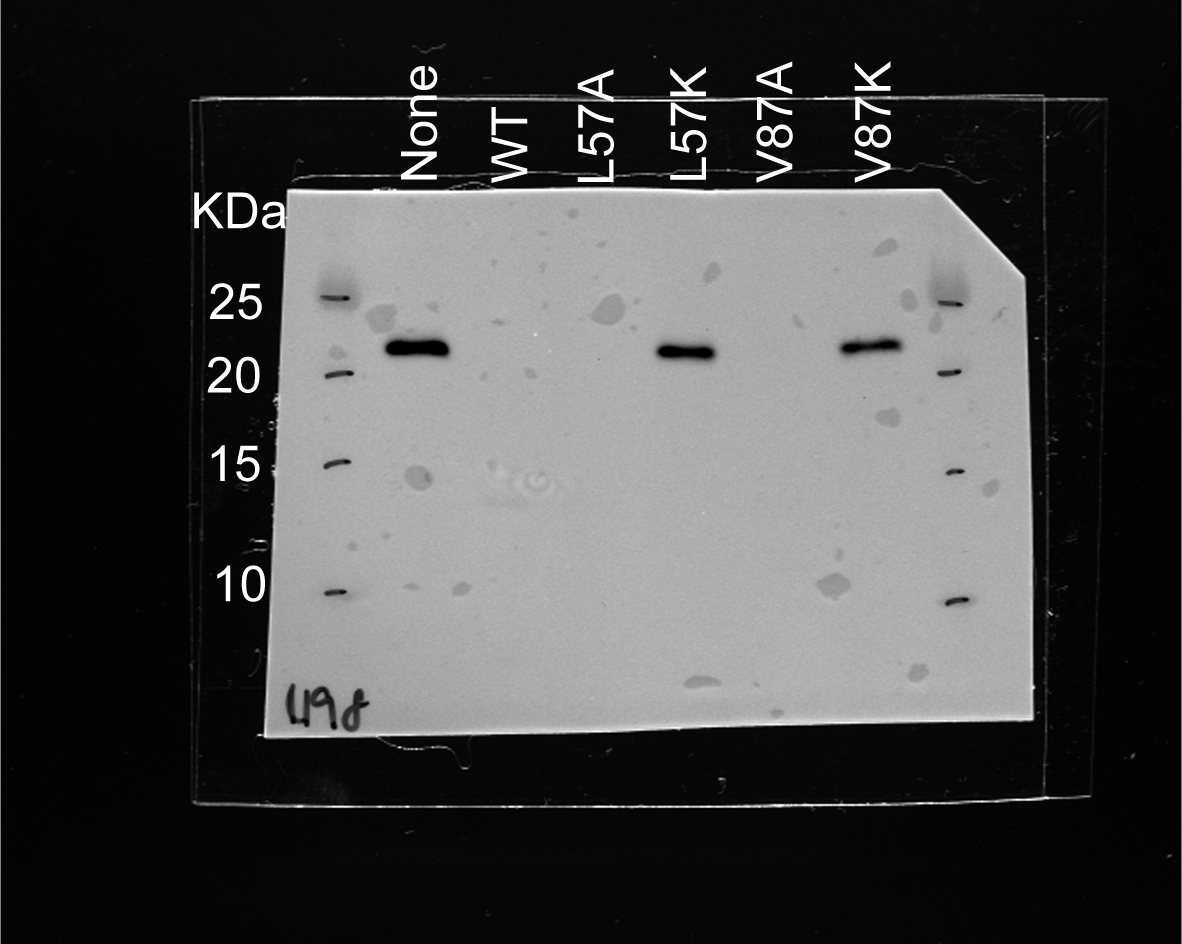

Supplement: Supplementary file 3 — Source Data Fig. 2 [file 44318_2024_69_MOESM3_ESM.zip › Figure-2/2i/LbcA mutants/PA1198 western ECL+visual overlay.tif]

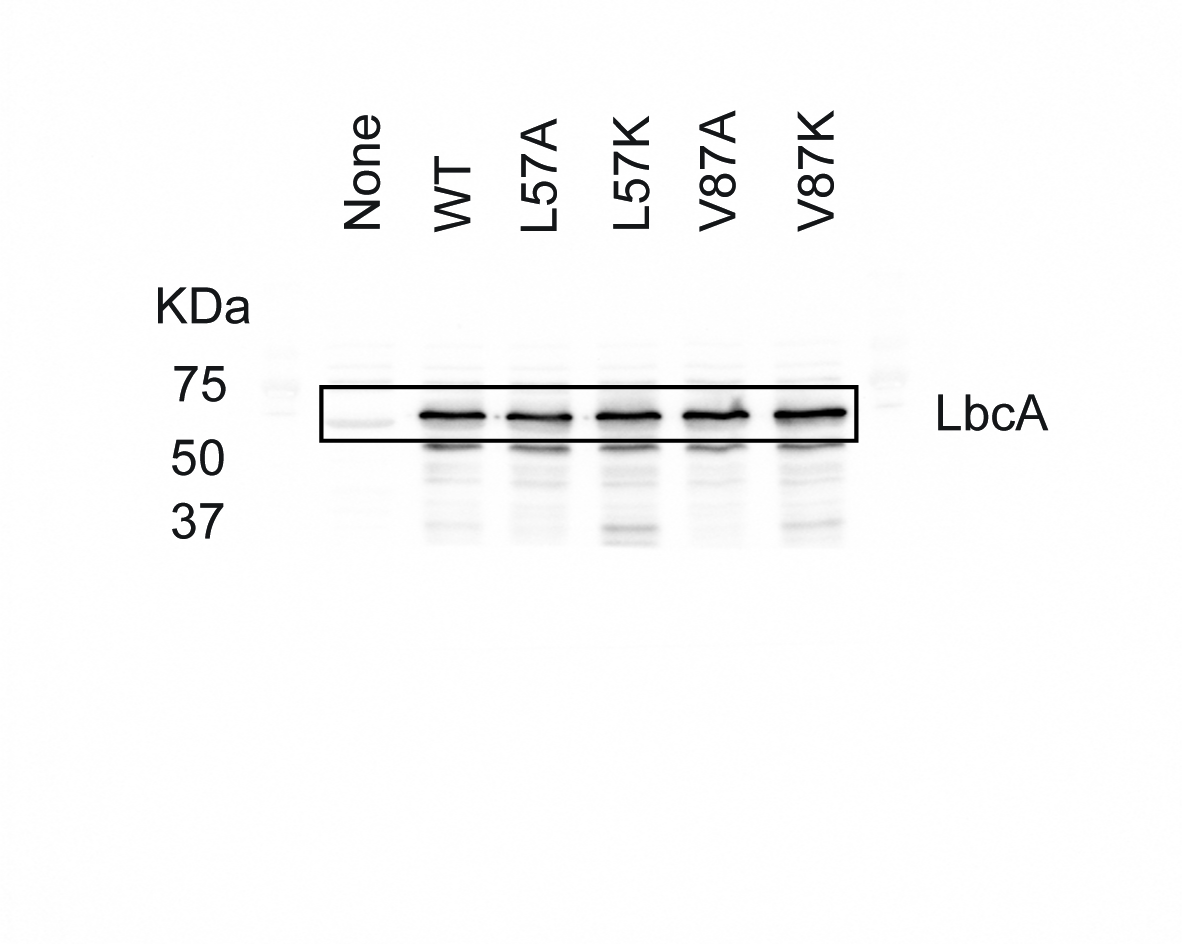

Supplement: Supplementary file 3 — Source Data Fig. 2 [file 44318_2024_69_MOESM3_ESM.zip › Figure-2/2i/LbcA mutants/LbcA western ECL only (used for figure).tif]

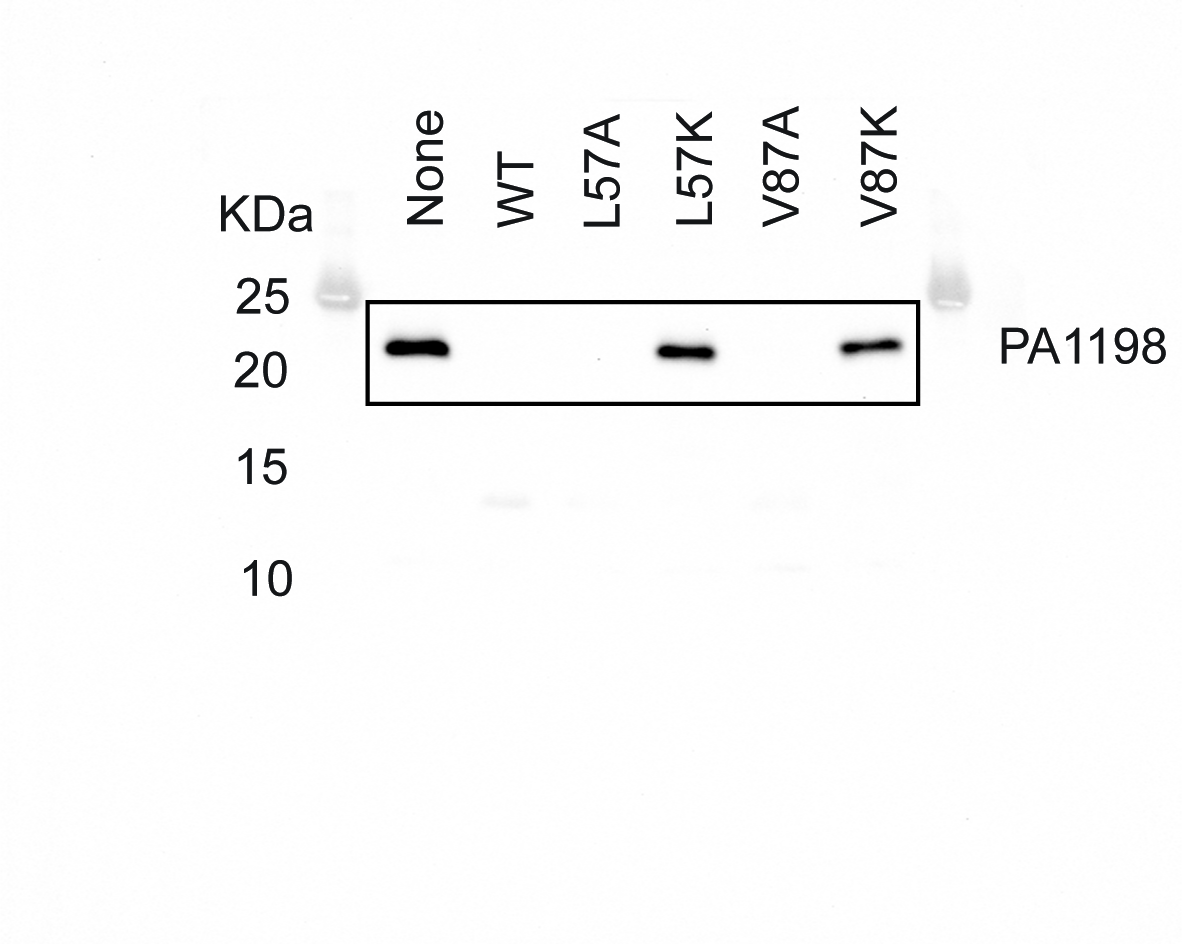

Supplement: Supplementary file 3 — Source Data Fig. 2 [file 44318_2024_69_MOESM3_ESM.zip › Figure-2/2i/LbcA mutants/PA1198 western ECL only (used for figure).tif]

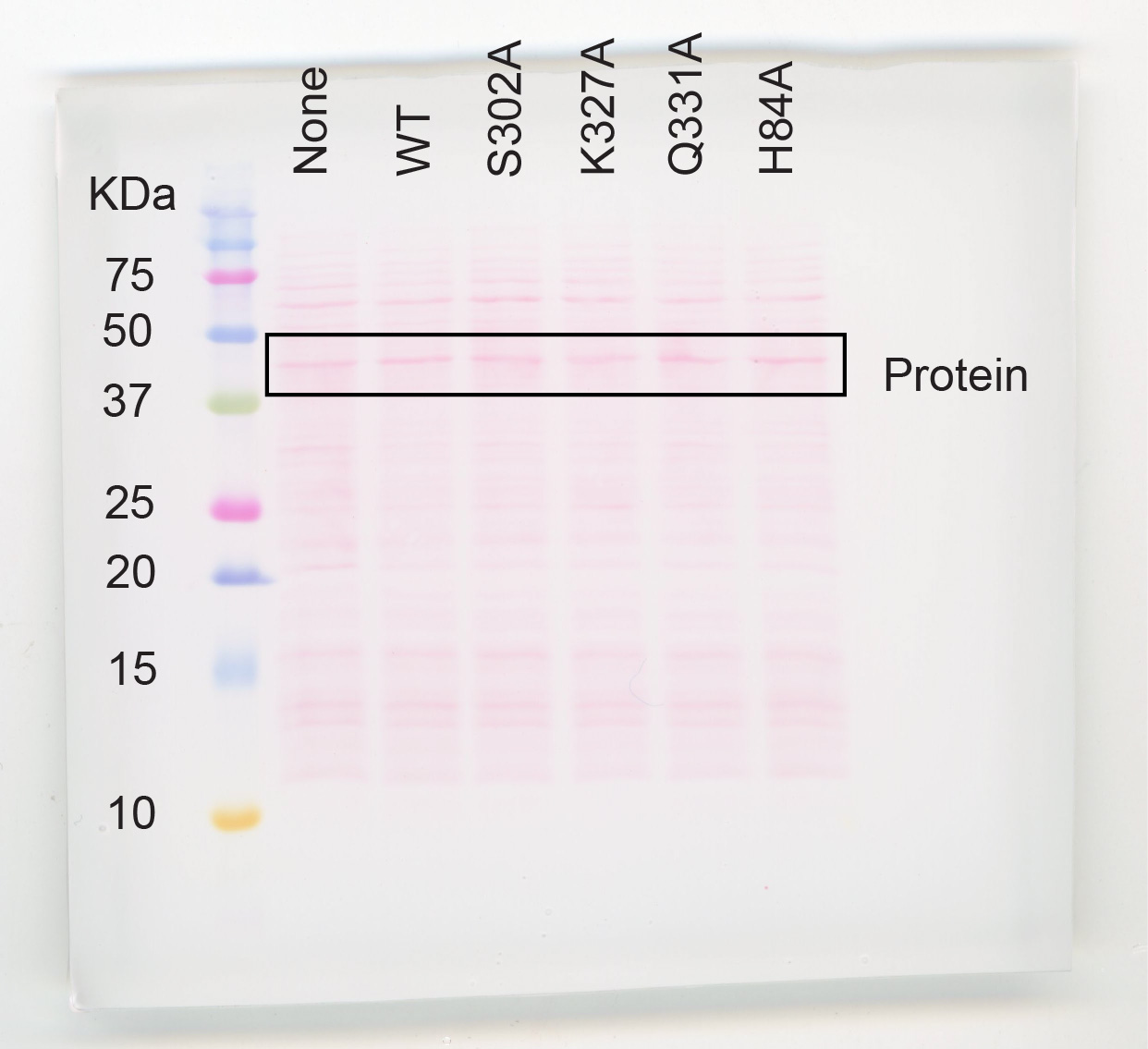

Supplement: Supplementary file 4 — Source Data Fig. 3 [file 44318_2024_69_MOESM4_ESM.zip › Figure-3/3h/Protein.jpg]

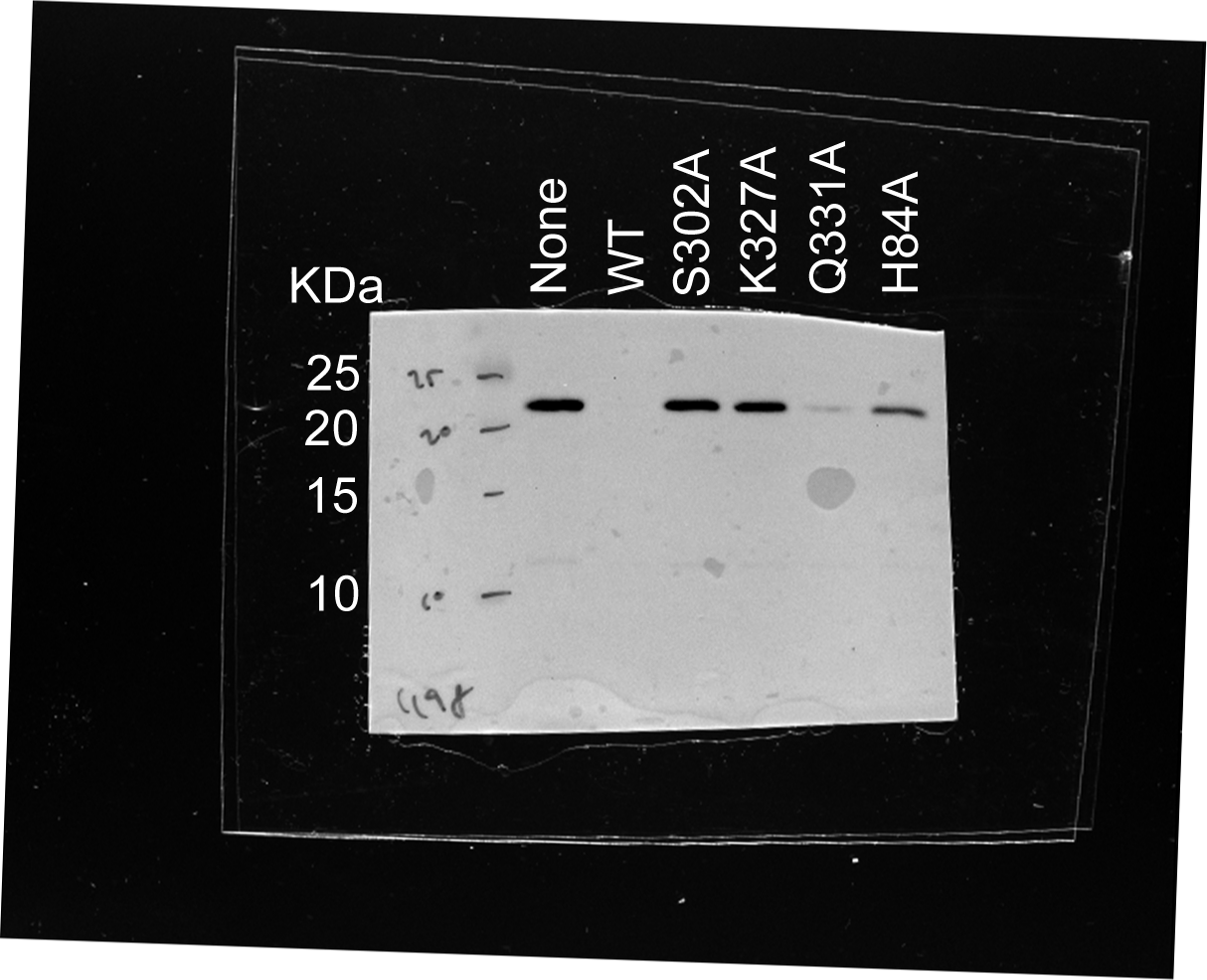

Supplement: Supplementary file 4 — Source Data Fig. 3 [file 44318_2024_69_MOESM4_ESM.zip › Figure-3/3h/PA1198 western ECL+visual overlay.tif]

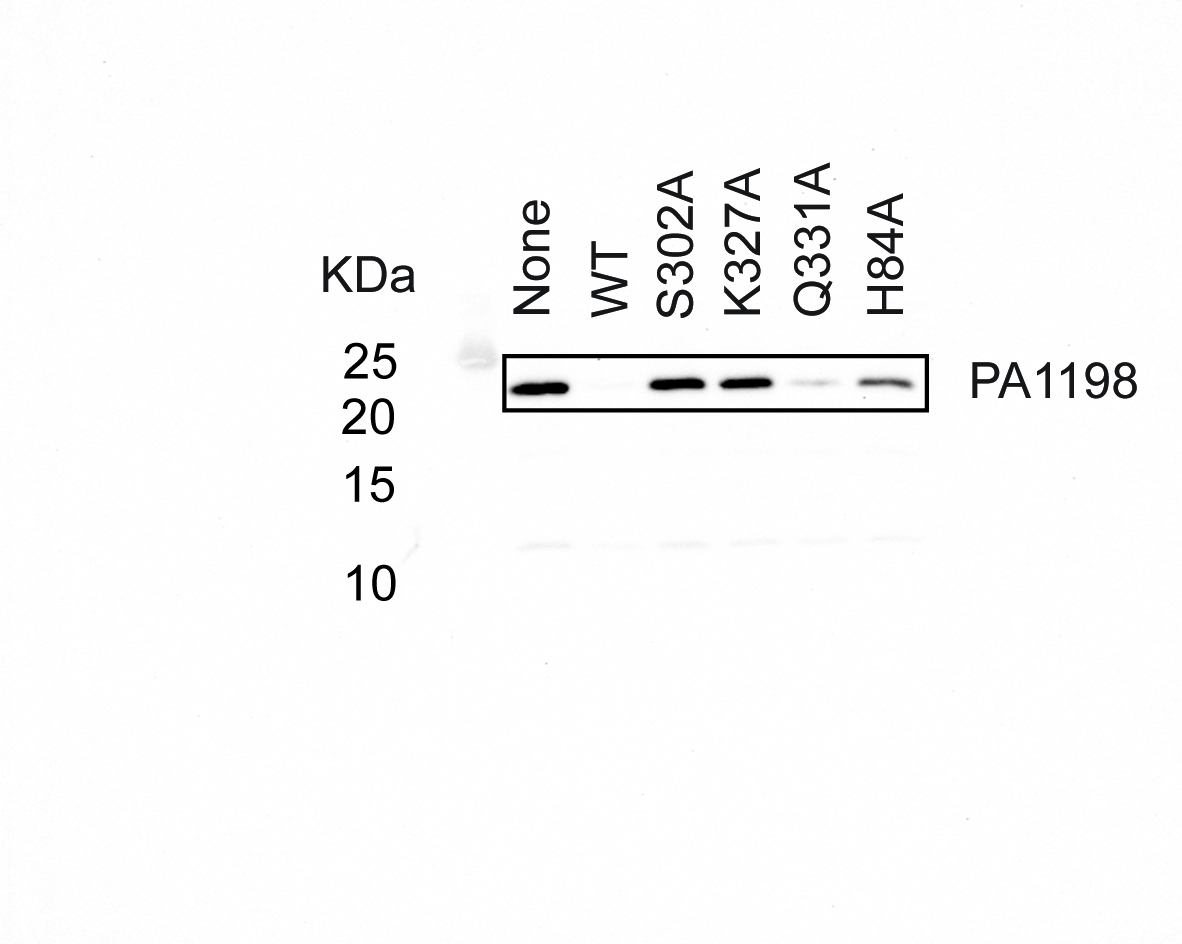

Supplement: Supplementary file 4 — Source Data Fig. 3 [file 44318_2024_69_MOESM4_ESM.zip › Figure-3/3h/PA1198 western ECL only (used for figure).tif]

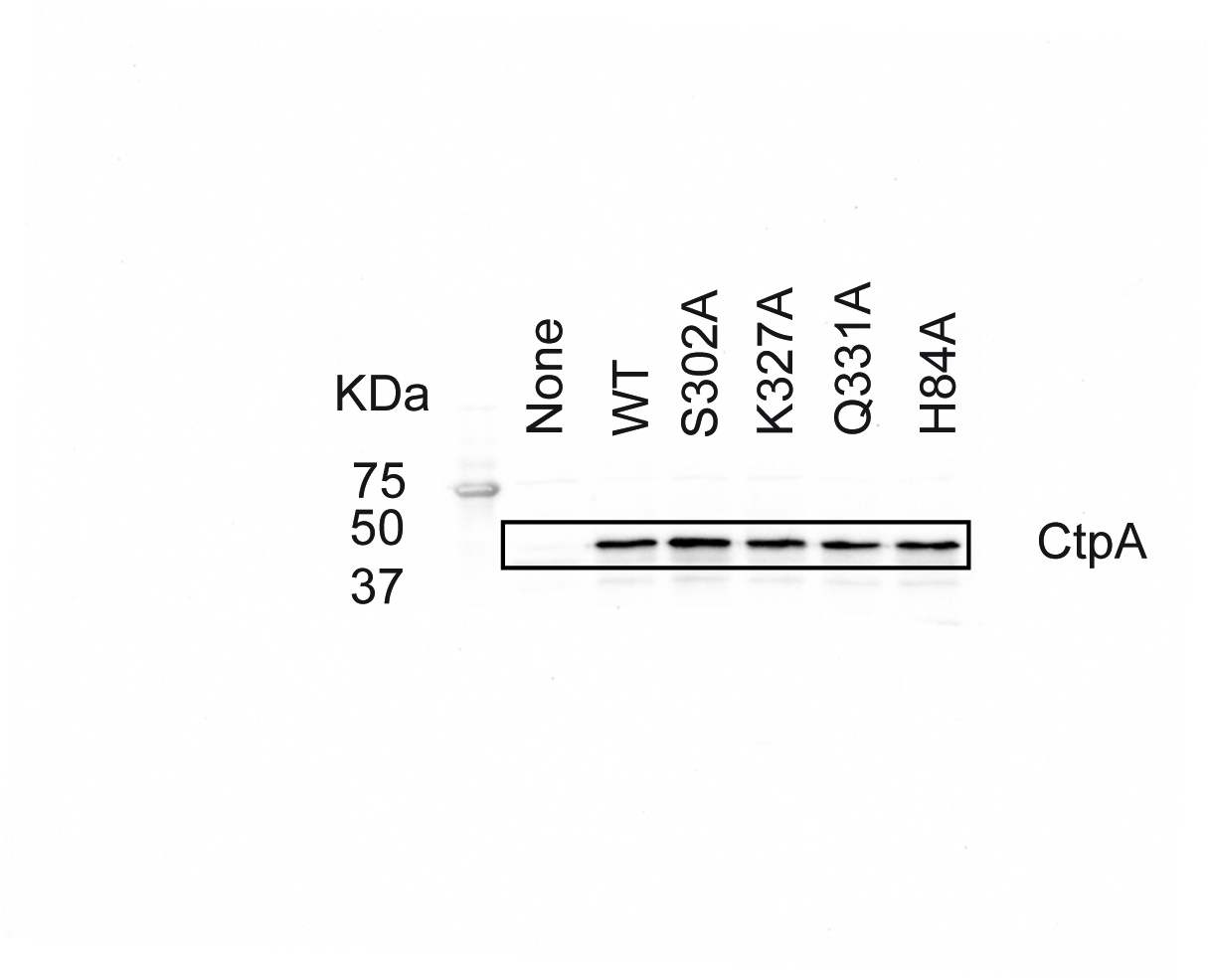

Supplement: Supplementary file 4 — Source Data Fig. 3 [file 44318_2024_69_MOESM4_ESM.zip › Figure-3/3h/CtpA western ECL only (used for figure).tif]

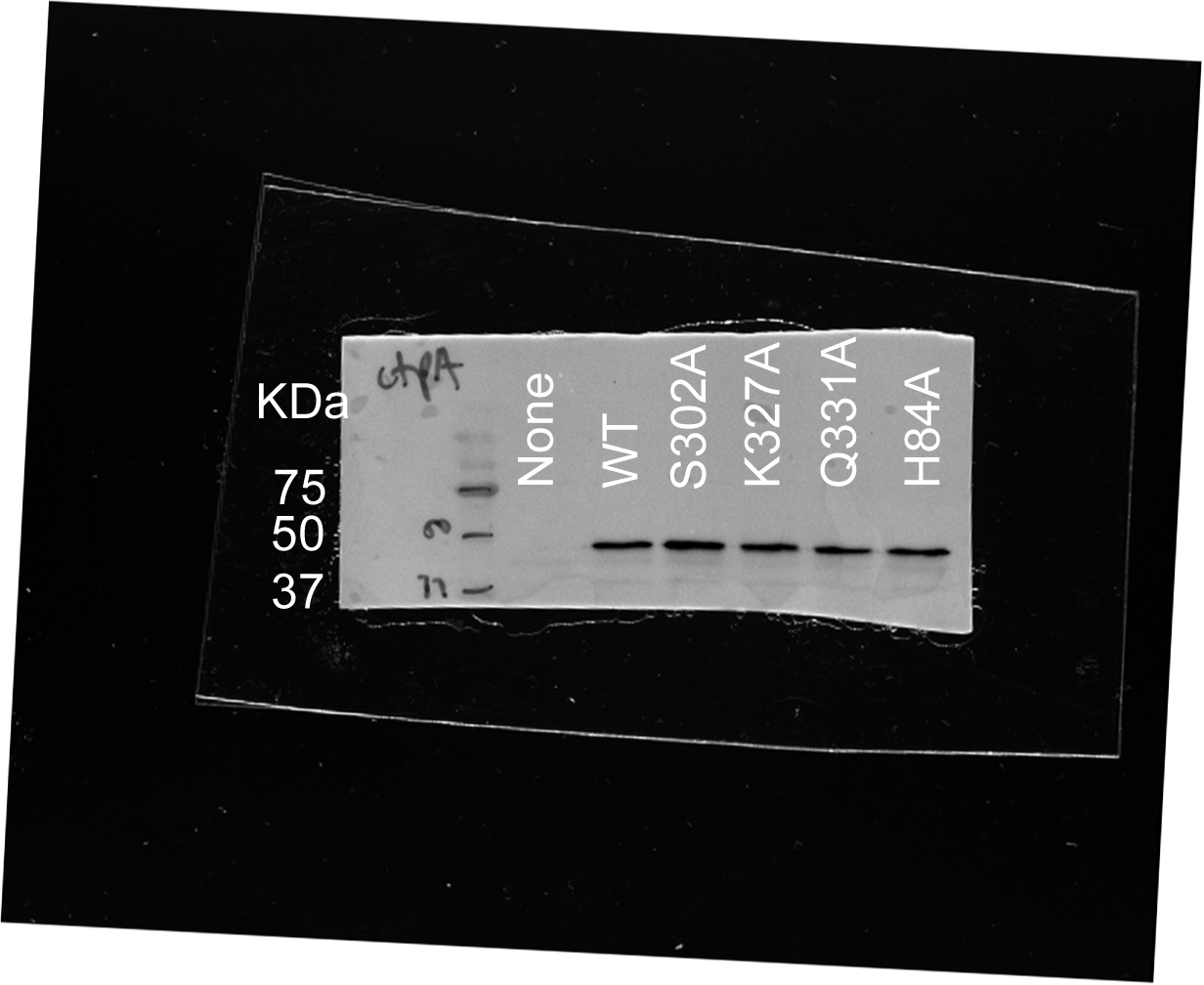

Supplement: Supplementary file 4 — Source Data Fig. 3 [file 44318_2024_69_MOESM4_ESM.zip › Figure-3/3h/CtpA western ECL+visual overlay.tif]

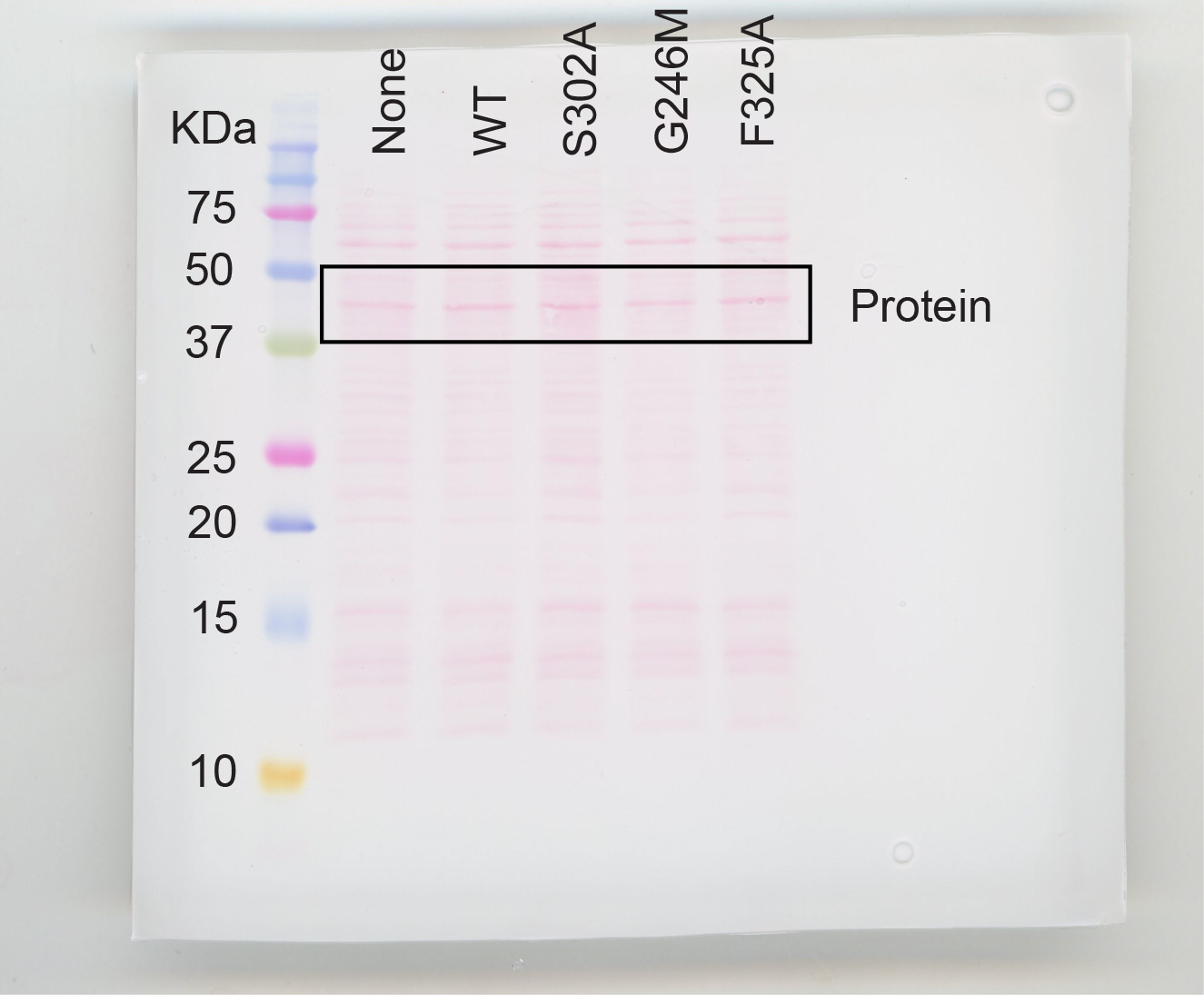

Supplement: Supplementary file 5 — Source Data Fig. 4 [file 44318_2024_69_MOESM5_ESM.zip › Figure-4/4e/Protein.jpg]

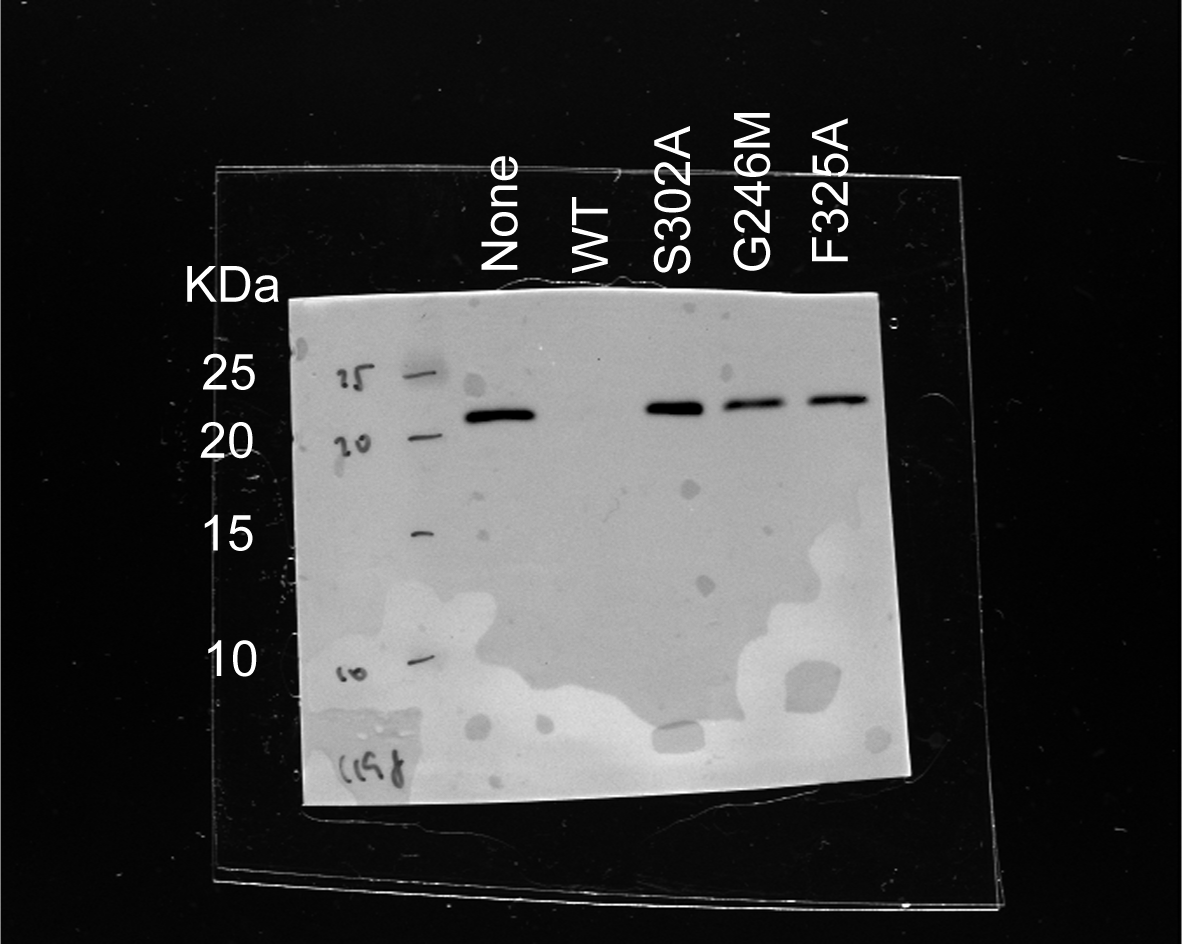

Supplement: Supplementary file 5 — Source Data Fig. 4 [file 44318_2024_69_MOESM5_ESM.zip › Figure-4/4e/PA1198 western ECL+visual overlay.tif]

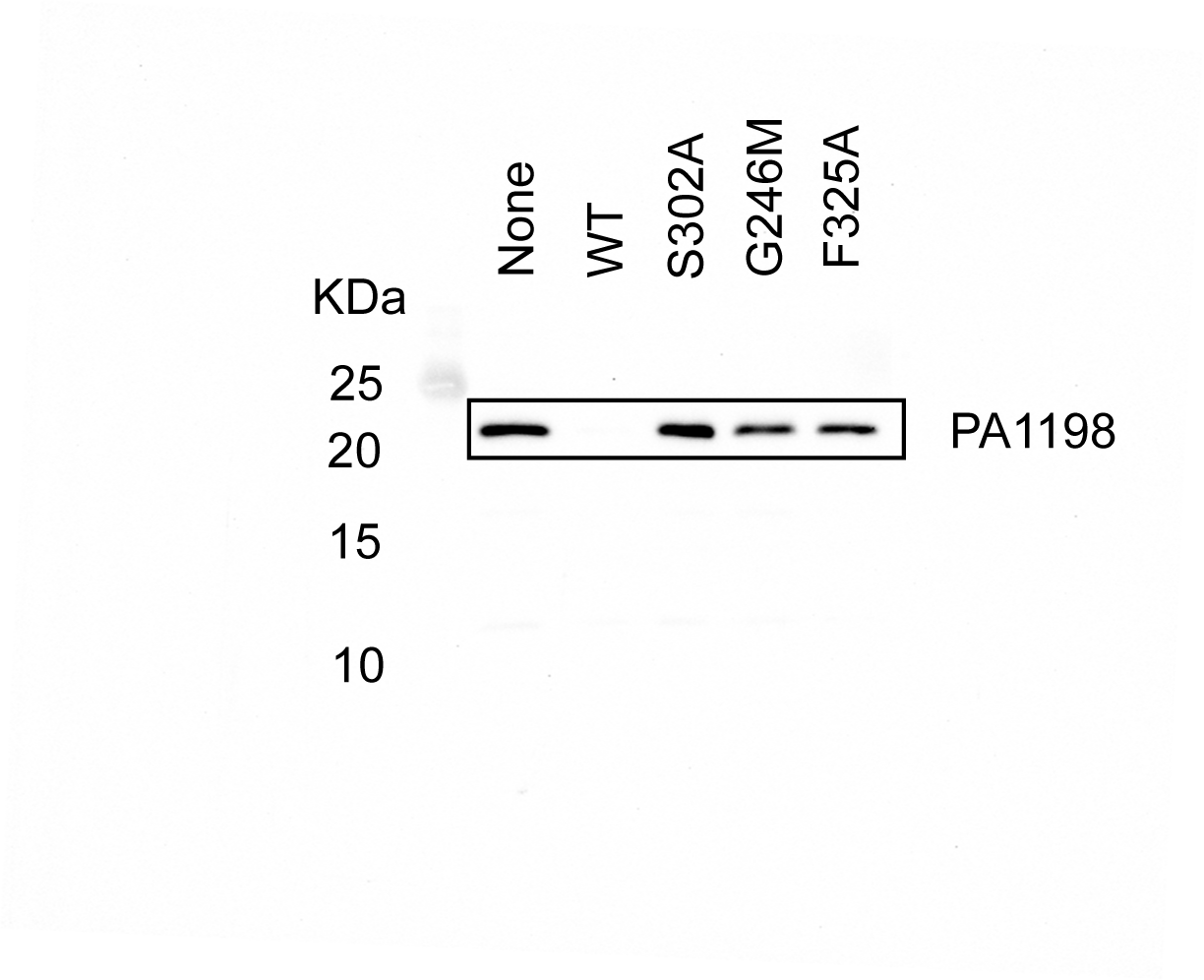

Supplement: Supplementary file 5 — Source Data Fig. 4 [file 44318_2024_69_MOESM5_ESM.zip › Figure-4/4e/PA1198 western ECL only (used for figure).tif]

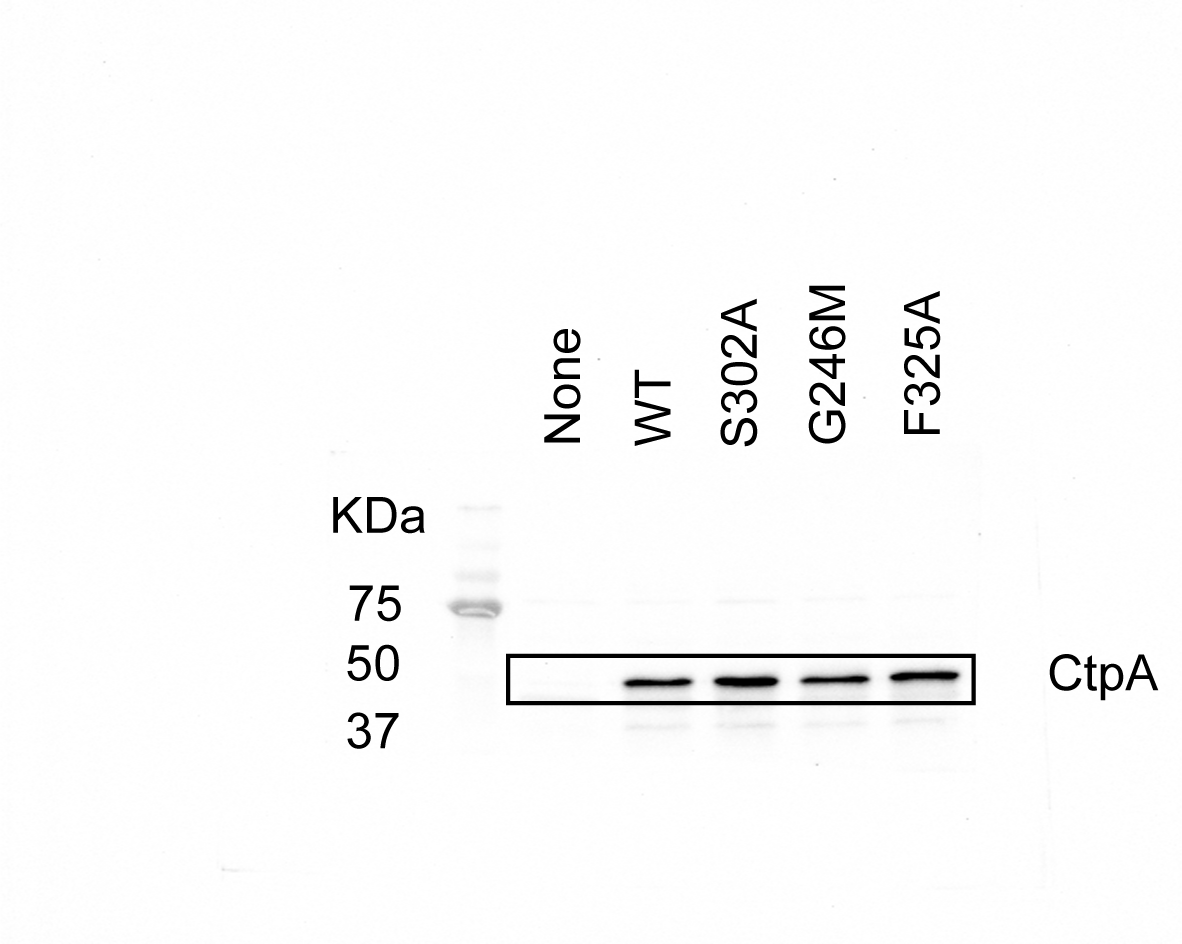

Supplement: Supplementary file 5 — Source Data Fig. 4 [file 44318_2024_69_MOESM5_ESM.zip › Figure-4/4e/CtpA western ECL only (used for figure).tif]

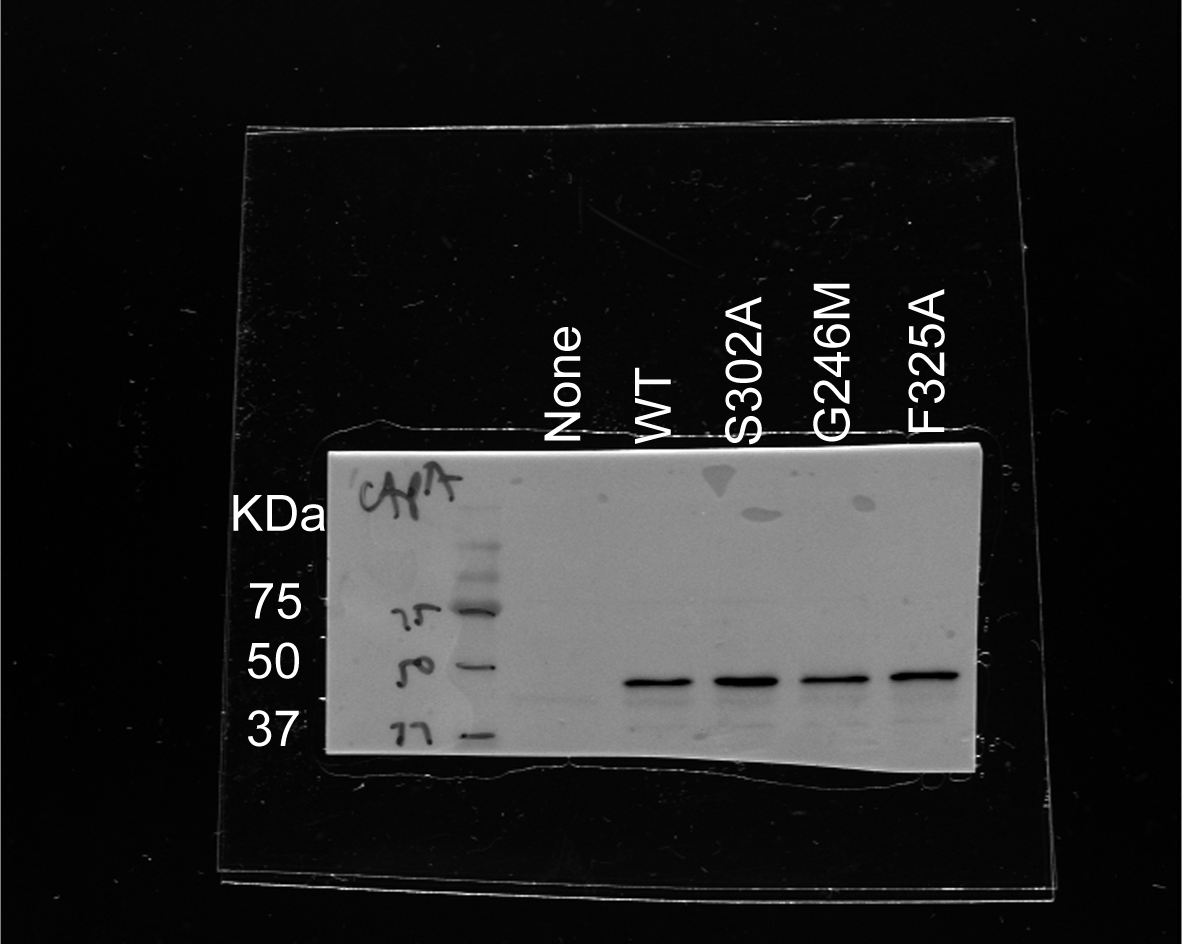

Supplement: Supplementary file 5 — Source Data Fig. 4 [file 44318_2024_69_MOESM5_ESM.zip › Figure-4/4e/CtpA western ECL+visual overlay.tif]

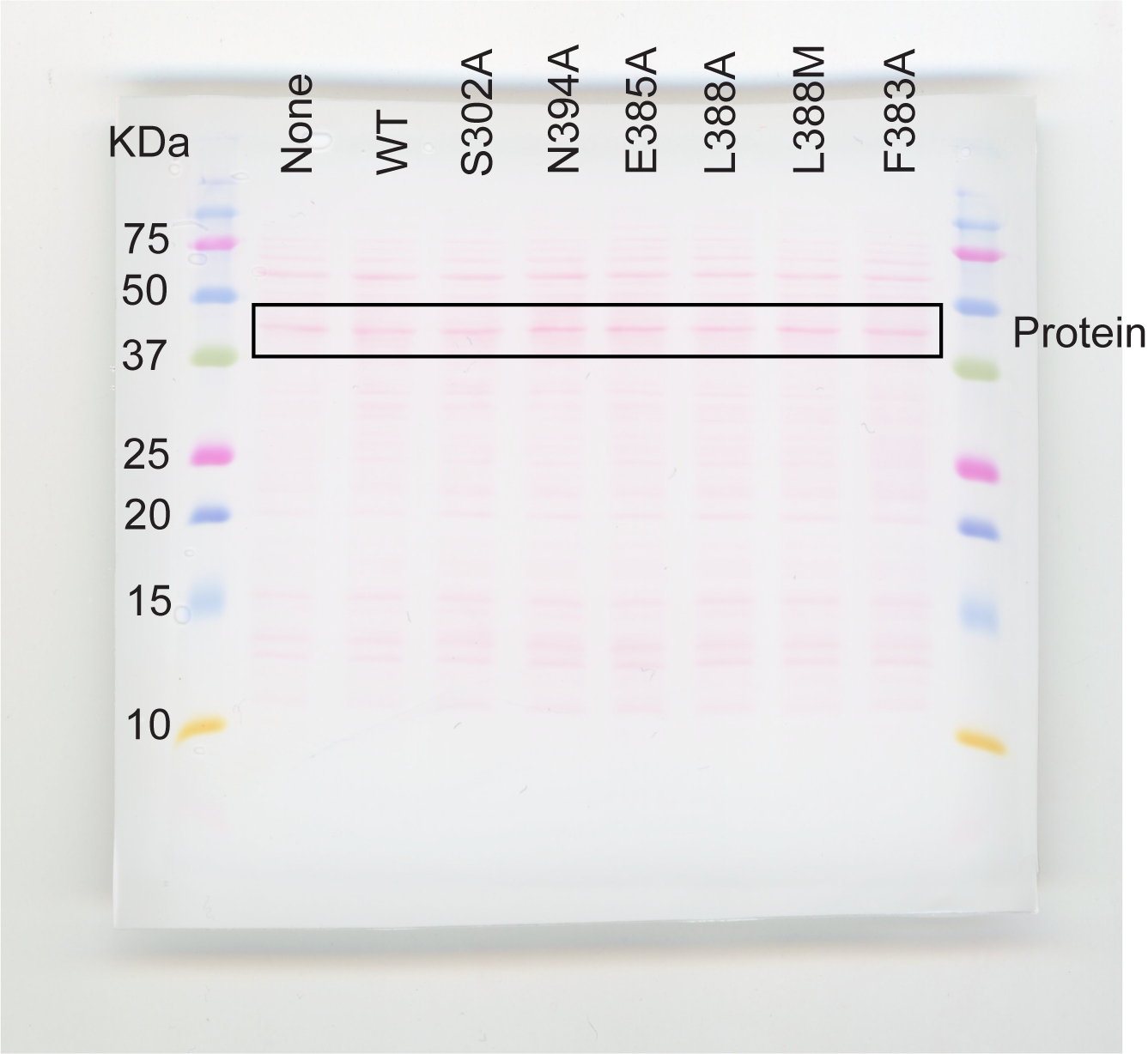

Supplement: Supplementary file 6 — Source Data Fig. 6 [file 44318_2024_69_MOESM6_ESM.zip › Figure-6/6f/Protein.tif]

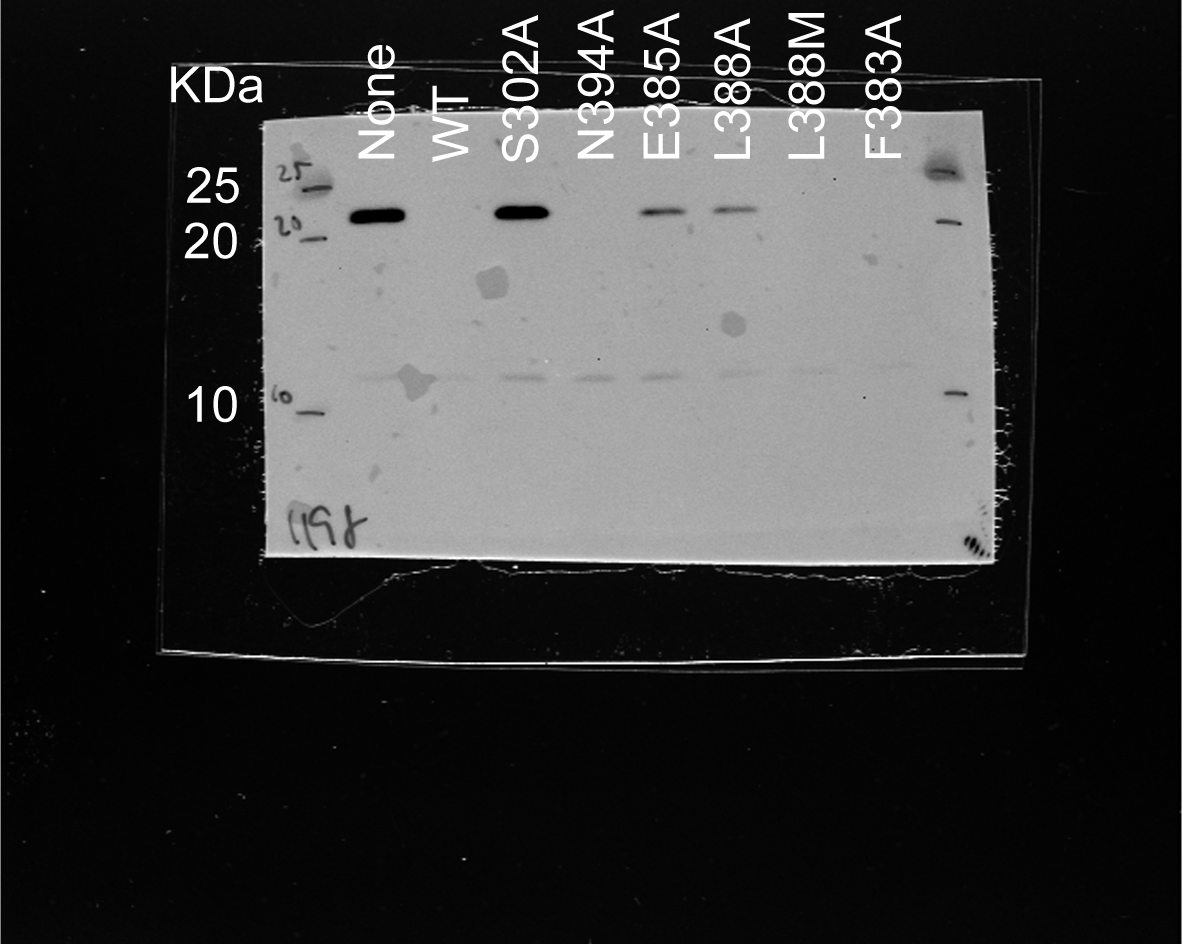

Supplement: Supplementary file 6 — Source Data Fig. 6 [file 44318_2024_69_MOESM6_ESM.zip › Figure-6/6f/PA1198 western ECL+visual overlay.tif]

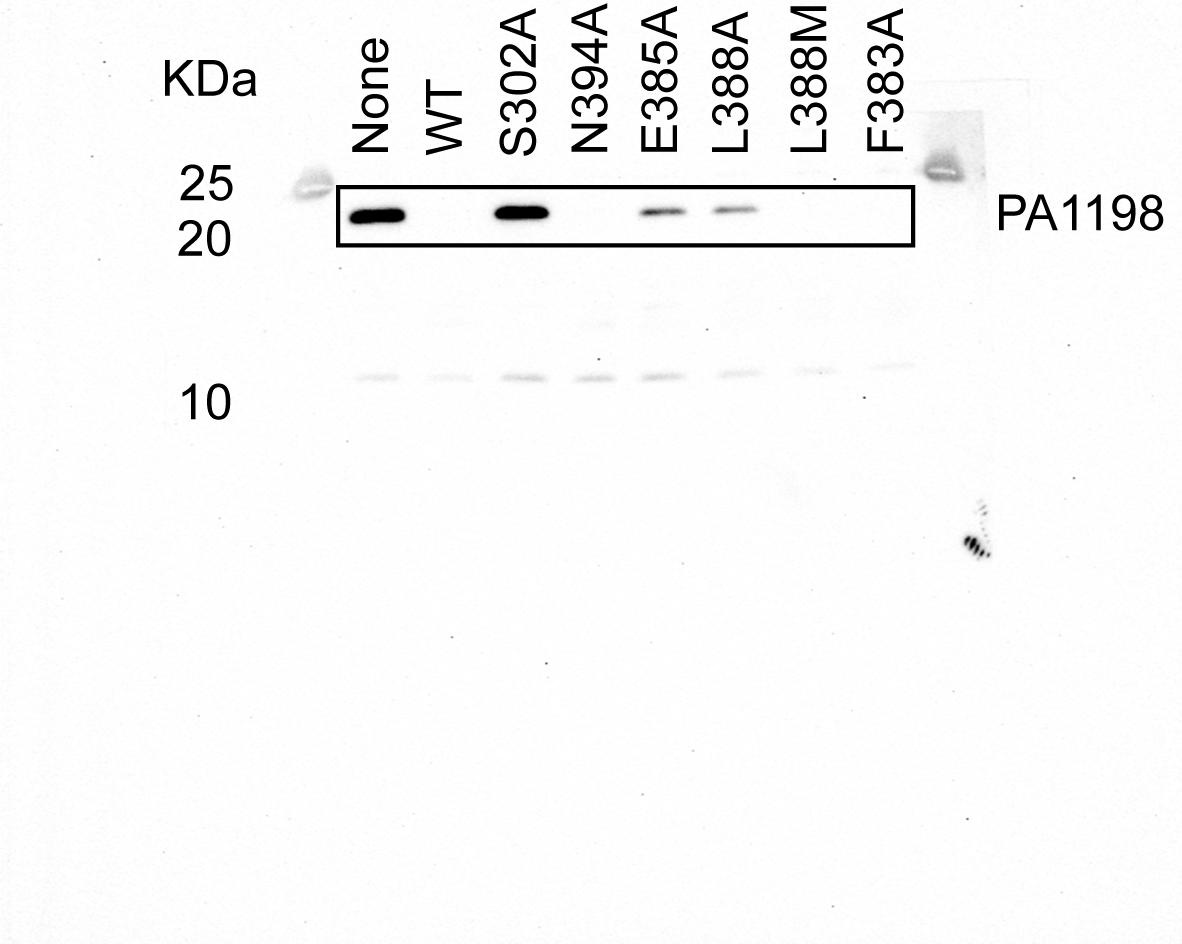

Supplement: Supplementary file 6 — Source Data Fig. 6 [file 44318_2024_69_MOESM6_ESM.zip › Figure-6/6f/PA1198 western ECL only (used for figure).tif]

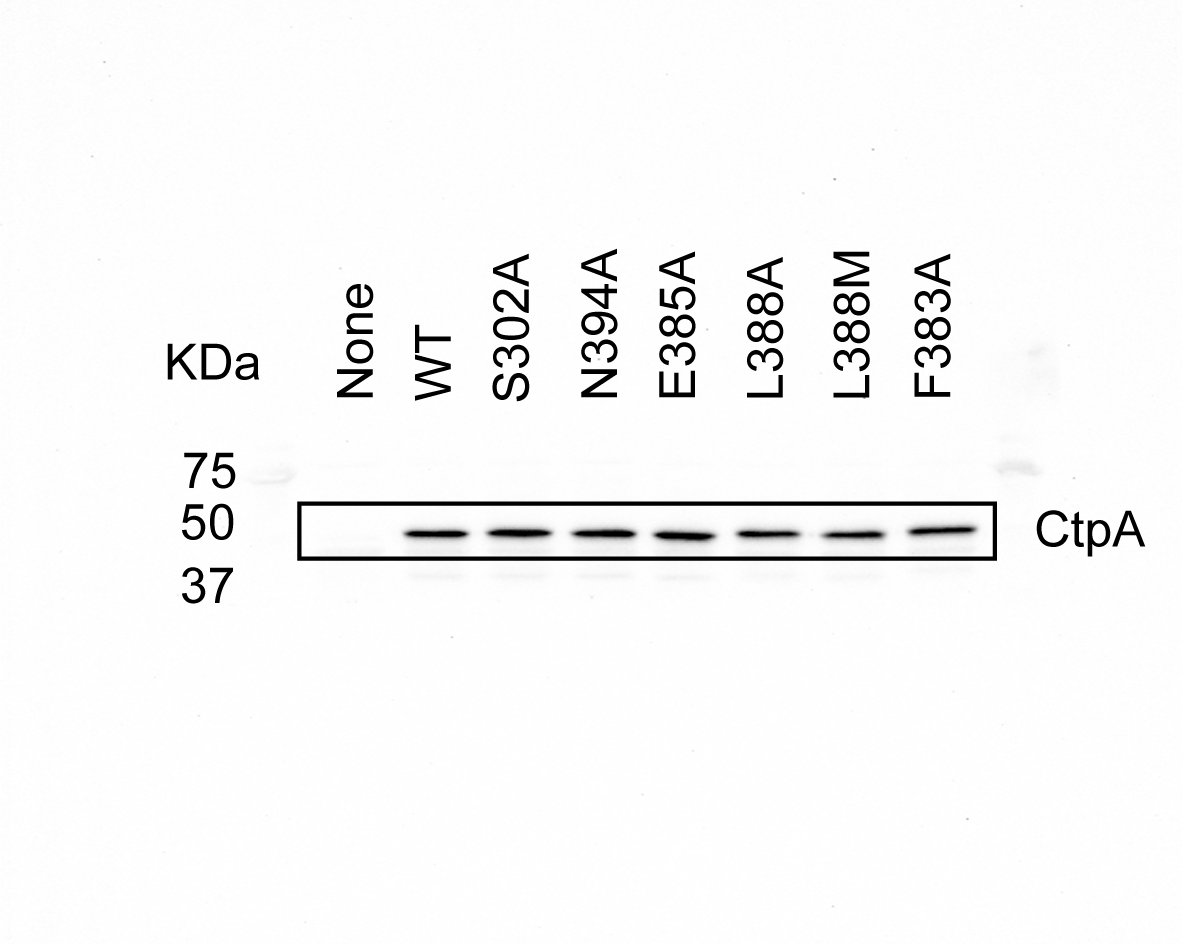

Supplement: Supplementary file 6 — Source Data Fig. 6 [file 44318_2024_69_MOESM6_ESM.zip › Figure-6/6f/CtpA western ECL only (used for figure).tif]

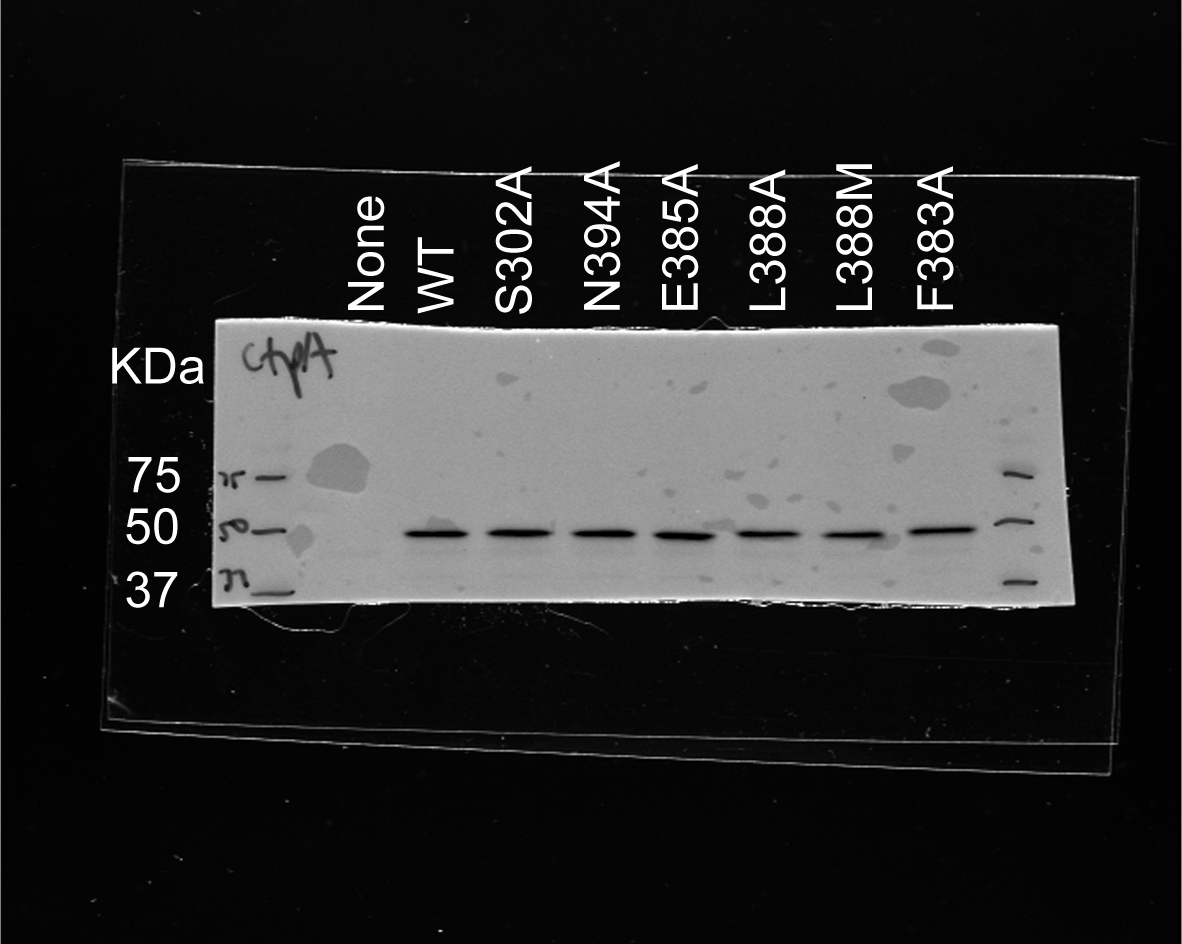

Supplement: Supplementary file 6 — Source Data Fig. 6 [file 44318_2024_69_MOESM6_ESM.zip › Figure-6/6f/CtpA western ECL+visual overlay.tif]

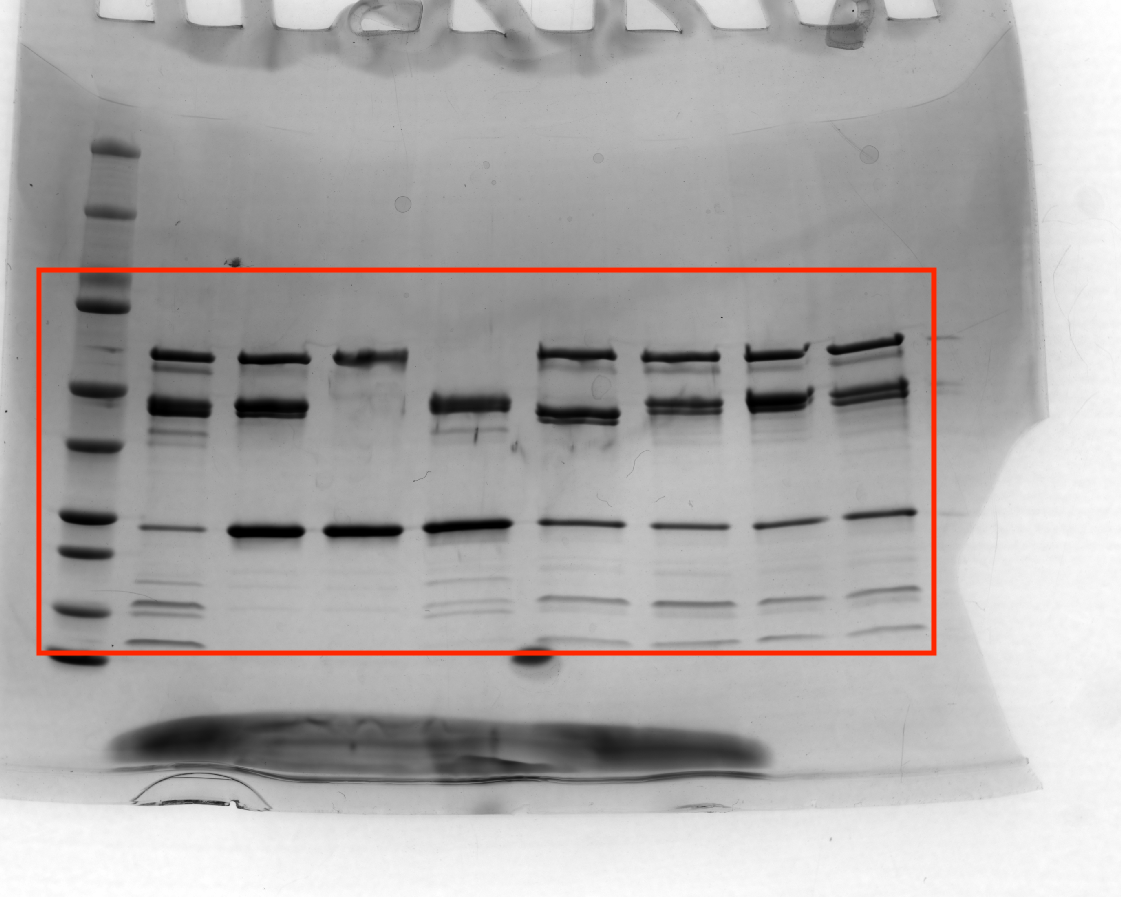

Supplement: Supplementary file 6 — Source Data Fig. 6 [file 44318_2024_69_MOESM6_ESM.zip › Figure-6/6g/Fig6g.tif]

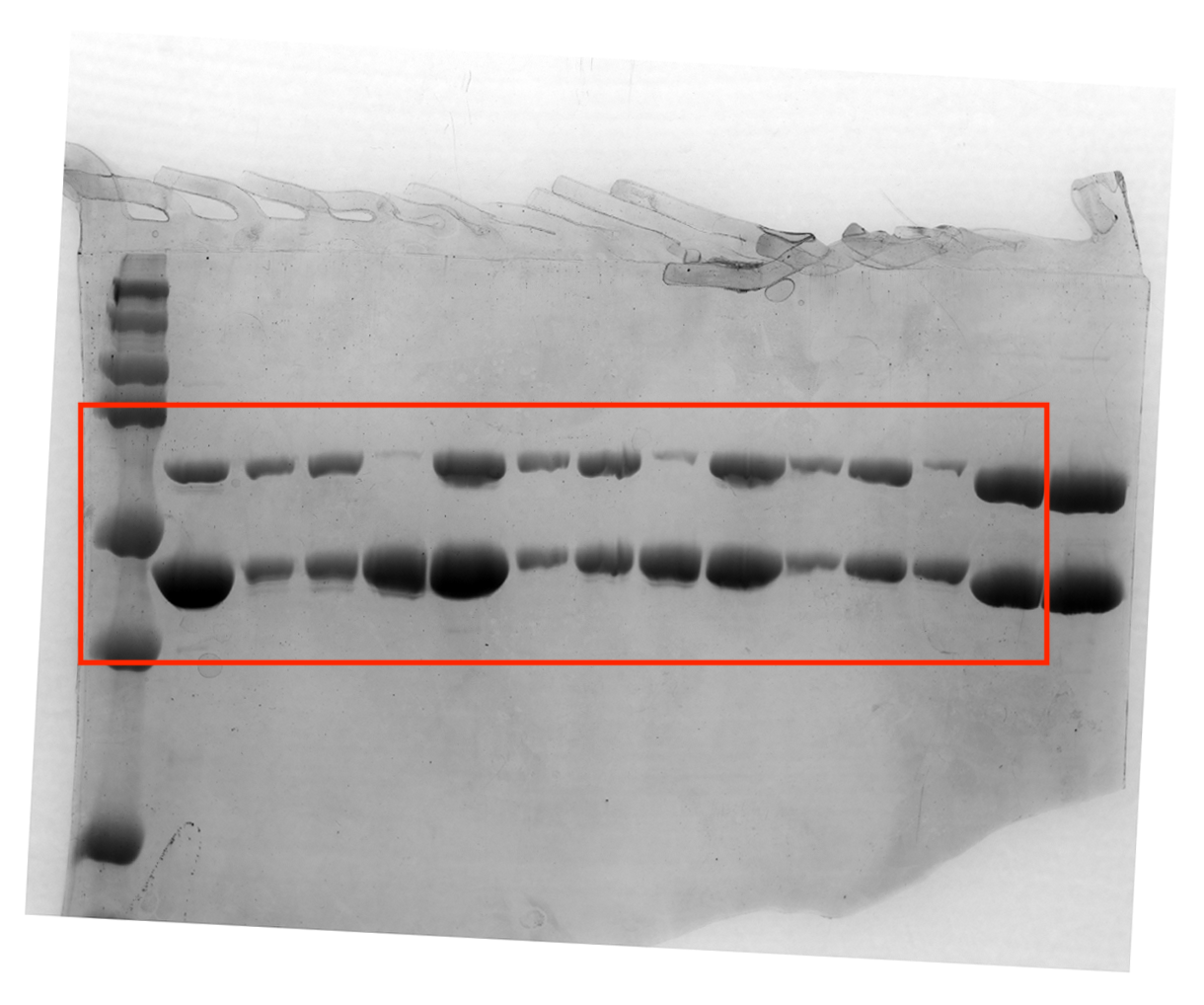

Supplement: Supplementary file 7 — Source Data Fig. 7 [file 44318_2024_69_MOESM7_ESM.zip › Figure-7/7d/7d.tif]
