# Supplementary material for: P. aeruginosa CtpA protease adopts a novel activation mechanism to initiate the proteolytic process
Source: EMBO J. 2024 Mar 11;43(8):1634–52. doi: 10.1038/s44318-024-00069-6 (PMC11021448; doi:10.1038/s44318-024-00069-6)
Supplement: Supplementary file 3 — Source Data Fig. 2 [file 44318_2024_69_MOESM3_ESM.zip › Figure-2/2j/Readme.pdf]

Bacterial two hybrid image

The brightness/contrast of the raw image was adjusted (for each entire plate) in order to improve visibility when used in the manuscript figure
