# Supplementary material for: P. aeruginosa CtpA protease adopts a novel activation mechanism to initiate the proteolytic process
Source: EMBO J. 2024 Mar 11;43(8):1634–52. doi: 10.1038/s44318-024-00069-6 (PMC11021448; doi:10.1038/s44318-024-00069-6)
Supplement: Supplementary file 3 — Source Data Fig. 2 [file 44318_2024_69_MOESM3_ESM.zip › Figure-2/2i/CtpA mutants/Readme.pdf]

For the CtpA and PA1198 immunoblots

The ECL only file was used to generate the figure.

The ECL+visual overlay image is also included because it also shows the visual appearance/size of the membrane piece used for each western.

Protein

This single membrane was used for both the CtpA immunoblot (top section) and PA1198 immunoblot (bottom section). Therefore, a single section as indicated was used from this membrane to show protein loading in the figure.
