# Supplementary material for: P. aeruginosa CtpA protease adopts a novel activation mechanism to initiate the proteolytic process
Source: EMBO J. 2024 Mar 11;43(8):1634–52. doi: 10.1038/s44318-024-00069-6 (PMC11021448; doi:10.1038/s44318-024-00069-6)
Supplement: Supplementary file 8 — Appendix Figure Source Data [file 44318_2024_69_MOESM8_ESM.zip › Source-Data-Appendix-Figure-S10.zip!/Appendix-Figure-S10/s10/reademe.rtf]

The rectangles were used for quantification.
