# Supplementary material for: P. aeruginosa CtpA protease adopts a novel activation mechanism to initiate the proteolytic process
Source: EMBO J. 2024 Mar 11;43(8):1634–52. doi: 10.1038/s44318-024-00069-6 (PMC11021448; doi:10.1038/s44318-024-00069-6)
Supplement: Supplementary file 8 — Appendix Figure Source Data [file 44318_2024_69_MOESM8_ESM.zip › Source-Data-Appendix-Figure-S6.zip!/Appendix-Figure-S6/S6b/Readme.pdf]

For the CtpA and PA1048 immunoblots

The ECL only file was used to generate the figure. The lower LbcA-specific bands are likely to be truncated LbcA proteins that become visible when LbcA is encoded on a plasmid. The most abundant band corresponding to full length LbcA was used for the figure, as boxed. In the PA048 immunoblot, the band above PA1048 is an unknown protein that is cross-reactive with the PA1048 polyclonal antiserum.

The ECL+visual overlay image is also included because it also shows the visual appearance/size of the membrane piece used for each western.

Protein

This single membrane was used for both the CtpA immunoblot (top section) and PA1048 immunoblot (bottom section). Therefore, a single section as indicated was used from this membrane to show protein loading in the figure.
